# A systematic review and meta-analysis assessing the influence of histone deacetylase 6 inhibition on brain infarction and neurological function following acute ischaemic stroke in rodent models

**DOI:** 10.1177/0271678X251405674

**Published:** 2026-01-18

**Authors:** Oliver B Ma, Timothy C Noack, Alexandra F. Trollope, Joseph V Moxon

**Affiliations:** 1 College of Medicine and Dentistry, James Cook University, Smithfield, QLD, Australia; 2 College of Medicine and Dentistry, James Cook University, Townsville, QLD, Australia; 3 Department of Anatomy and Pathology, College of Medicine and Dentistry, James Cook University, Townsville, QLD, Australia; 4 Australian Institute of Tropical Health and Medicine, James Cook University, Townsville, QLD, Australia

**Keywords:** Ischemic stroke, histone deacetylase 6, meta-analysis, cytoprotection, functional recovery

## Abstract

Histone deacetylase (HDAC) inhibition has been suggested to improve stroke outcomes, however, pan-HDAC inhibition can cause adverse effects. Individual studies report that specifically inhibiting HDAC6 may improve stroke outcomes. This article aimed to quantify the impact of pharmacologically inhibiting HDAC6 on cerebral infarction size and neurological function within *in vivo* stroke models. Seven studies fulfilled inclusion criteria. Meta-analysis demonstrated that animals receiving HDAC6 inhibitors developed significantly smaller cerebral infarctions than controls (standardised mean difference −1.25, 95% confidence intervals: −1.68, −0.81). This was upheld in sub-analyses comparing different species, and groups receiving HDAC6 inhibitors shortly after stroke, or after a 24 h delay. Modelling analyses demonstrated that animals receiving HDAC6 inhibitors during the hyperacute phase of stroke had significantly better functional outcomes, whereas sub-acute HDAC6 inhibition impaired recovery. Despite a small evidence base, findings suggest that HDAC6 inhibition within 24 h of stroke onset may improve outcomes. Current understanding is limited by male bias in existing studies and a lack of assessment in models incorporating stroke comorbidities or risk factors. Studies employing randomised controlled trial principles, and detailed assessment of the molecular and physiological mechanisms underpinning reported cytoprotection are warranted to assess clinical potential pharmacological HDAC6 inhibition to improve stroke management.

## Introduction

An estimated 11.9 million people experience stroke each year, 65%–80% of which occur due to a disruption of cerebral blood flow resulting in an area of focal ischaemia (acute ischaemic stroke (AIS)).^1,2^ Following AIS critically hypo-perfused brain tissue rapidly necroses forming an ischaemic core bordered by an ischaemic penumbra comprising poorly perfused but viable tissue. With prolonged poor perfusion the ischaemic penumbra progressively necroses, causing the ischaemic core to expand.^3^ AIS therapies therefore focus on restoring local blood flow to reperfuse and salvage the ischaemic penumbra and reduce the extent of irreversible damage to the brain but are only indicated for use within hours of symptom onset.^4,5^ The high prevalence of unrelated conditions mimicking the non-specific neurological symptoms of AIS,^6^ and risks of fatal bleeding associated with pharmacological reperfusion means that a definitive AIS diagnosis must be established via brain imaging and specialist assessment before commencing treatment.^4,5,7^ This contributes to a disparity in health outcomes for rural patients who may incur time penalties to care provision whilst in transit to these facilities.^8,9^ Patient advocates and end users have therefore stated that identifying cytoprotective agents to preserve at-risk tissue prior to reperfusion, particularly for individuals requiring transportation, as a key research priority.^10,11^

Histone deacetylases (HDACs) are a class of enzymes (EC 3.5.1.98) which post-translationally remove acetyl groups from protein targets.^12,13^ Original investigations identified a role for the HDACs in epigenetic modification of histones, however, an increasing number of non-histone targets have also been described (reviewed by Park et al.,^13^ Curcio et al.,^14^ and Xu et al.^15^). Increasing evidence highlights a potential role for the HDACs in the response to AIS. For example, genome-wide association studies have highlighted that a single nucleotide polymorphism (RS11984041) in the *HDAC9* gene is associated with increased risk of large artery AIS.^16,17^ *In vitro* and *in vivo* data have also highlighted that the expression and activity of multiple HDACs is increased in AIS-affected brain tissues.^18–20^ Independent studies using *in vitro* and *in vivo* models have also reported that AIS-severity is significantly reduced when administering pharmacological HDAC inhibitors, compared to controls (reviewed by Majdi et al.^21^). Observational cohort studies have also identified that participants receiving the anti-seizure medication sodium valproate, a non-specific HDAC inhibitor, exhibited significantly lower risks of experiencing AIS than those receiving other anti-epileptic medications.^22,23^ Inhibiting multiple HDACs has potential to cause serious adverse effects due to the role for this enzyme family in homeostatic pathways including cell cycle regulation, inflammation and mitochondrial function and it has been suggested that these aberrant effects may be reduced by inhibiting specific HDAC family members.^13,14,24,25^

HDAC6 is a class IIb member of the HDAC family which has been shown to be significantly over-expressed within the ischaemic penumbra following experimental AIS.^19,26^ Reports of favourable outcomes following HDAC6 inhibition for multiple neurological indications suggests strong potential for this protein to act as a cytoprotective target.^27–29^ A recent meta-analysis reported that HDAC6 inhibition was associated with smaller cerebral infarction sizes in rodent models of AIS, but did not investigate the impact of HDAC6 inhibition on functional outcomes.^21^ The current article aimed to address this gap in knowledge by comprehensively assessing all available evidence on the impact of HDAC6 inhibition on the severity of AIS pathology and neurological function during recovery in experimental models.

## Methods

This systematic review was performed in line with the PRISMA Guidelines and the Systematic Review Centre for Laboratory animal Experimentation statement.^30,31^ An overarching systematic review protocol for an article assessing the impacts of HDAC inhibition on AIS outcomes was registered with the PROSPERO database (CRD420250643034). Literature searches confirmed that the scale of this work was beyond the scope of an individual study and a protocol amendment reflecting the current focus on HDAC6 was submitted to PROSPERO.

### Literature searching

**Figure 1.**
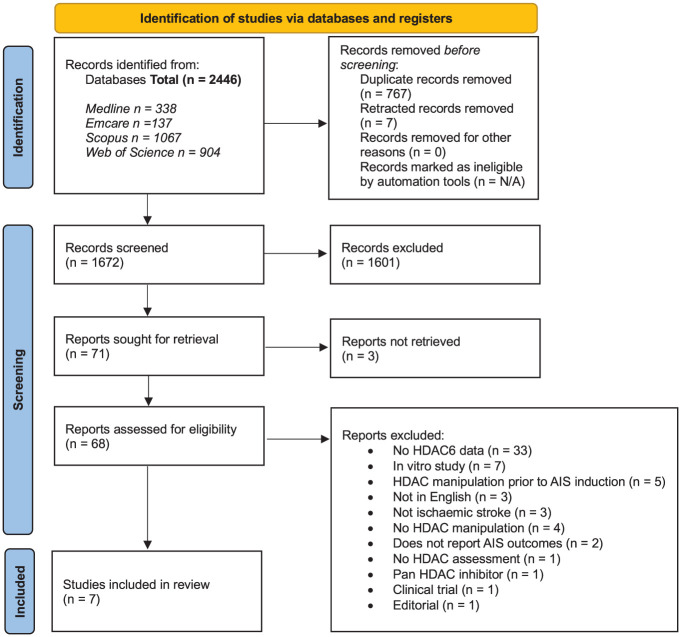
PRISMA diagram detailing the article selection process for this review.

A systematic literature search was conducted using a search strategy designed in collaboration with a specialist librarian (Supplement 1, Figure 1). Retrieved articles were screened by four authors (OBM, TCN, JVM, AFT) to identify relevant studies. Studies eligible for inclusion were required to: (i) utilise *in vivo* models of focal AIS; (ii), administer inhibitors with demonstrated ability to specifically inhibit HDAC6 to the experimental group and (iii) report outcomes relating to AIS pathology or neurological outcomes relative to an appropriate control group. Studies that assessed pan-HDAC inhibitors, or drug cocktails with pleiotropic effects were excluded, unless data relating to the specific effects of inhibiting HDAC6 were also presented. Articles written in languages other than English, review articles, editorials, commentaries and conference abstracts which did not provide experimental data were also excluded. To mimic the clinical scenario, data from studies that administered *HDAC6* inhibitors prior to AIS induction (including organisms with a genetic modification to alter HDAC6 activity) were not eligible for inclusion. The final literature search was performed on 21st March 2025.

### Data extraction and risk of bias assessment

Three authors (OBM, TCN, JVM) independently performed data extraction and assessed the risk of bias of included studies. Discrepancies were addressed at a consensus meeting involving a fourth author (AFT) as arbitrator. Extracted information included details of the animal model used (species, strain, age/body weight and sex), method of AIS induction, duration of cerebral ischaemia, and the time of sacrifice relative to AIS induction. Key details collected regarding the HDAC6 inhibition strategy included the drug name, dose, route of administration, timing and frequency of administration relative to AIS onset, duration of the experiment and approaches used to confirm the ability of the agent to inhibit HDAC6 *in vivo*. Methods used to assess outcomes (e.g. infarction size, neurological function) and the timing of assessment relative to AIS induction (defined as hyperacute (0–6 h), late hyperacute (6–24 h), acute (24 h–7 days), or sub-acute phases (7 days–3 months) were documented.^3,32,33^ Reported numerical data were extracted; where necessary, data were interpolated from presented graphs by three independent authors (OBM, TCN and JVM) using the image analysis function of Photoshop 2024 (Adobe Inc., San Jose, CA, USA). If this was not possible study authors were contacted to provide additional data. The risk of bias of included studies was assessed using a modified CAMARADES score as previously described (Supplement 2).^3,34,35^ AIS-relevant questions based on Stroke Treatment Academic Industry Roundtable (STAIR) recommendations and whether authors had clearly demonstrated that the employed drugs had successfully inhibited HDAC6 *in vitro* or *in vivo* were also inserted (Supplement 2).^36,37^ Scores for each study were calculated based on the number of ‘yes’ answers to the posed questions. Each study could receive a maximum score of 13; scores of <4, 5–6 or >7 were considered to denote high, medium or low risks of bias, respectively.

#### Meta-analyses assessing the effects of *HDAC6* inhibitors on cerebral infarction size

The R meta and dmetar packages were used to generate random-effects models estimating the standardised mean differences in cerebral infarction sizes of animals receiving *HDAC6* inhibitors or controls.^38,39^ As all studies reported outcomes as mean and standard error of the mean (SEM) standard deviations were calculated by multiplying the SEM by the square root of the sample size as previously described.^34,40^ For studies comparing multiple experimental groups to a common control group, the number of control animals was split to provide groups with equal mean and SD in order to provide each intervention with a comparable control whilst avoiding double-counting (as per Cochrane Collaboration recommendations and our prior meta-analyses).^34,41^ Leave-one-out analyses were conducted to investigate the influence of individual studies on the overall effect size. Funnel plots and Egger’s regression were used to assess publication bias. Trim and fill analyses were used to estimate the number of additional datasets to achieve perfect symmetry in funnel plots – these were then imputed and models rerun to assess their impact on the observed effects. Subgroup analyses investigated whether the effect of *HDAC6* inhibition on infarction size was influenced by the animal species used or the timing of drug administration relative to AIS induction (defined as rapid (within 1 h of PTI or upon reperfusion for MCAO models) or delayed (>24 h post-AIS)). Secondary analyses assessed the influence of (i) commercially available *HDAC6* inhibitors and (ii) the most commonly administered *HDAC6* inhibitor, on brain infarct size. Meta-regression was conducted to investigate whether there was any association of the number of drug doses administered, or the delay between AIS induction and tissue harvest with observed cerebral infarction size.

### Meta-analysis of longitudinal data

Several studies presented continuous longitudinal datasets assessing the recovery of forelimb function (presented by Demyanenko et al.^18,19^), rotarod performance and body tilt data^42^ in separate experiments and did not directly compare the relative efficacy of different *HDAC6* inhibition regimes on these outcomes. As raw data were not available for these experiments, 15,000 simulated datasets approximating the reported means and SEMs from the original reports were generated (*n* = 8/group/simulation as per the original sample sizes. Supplements 8 and 9). Left forelimb use (provided by Demyanenko et al.^18,19^) and body tilt data^42^ were reported as percentages. Data simulations for these outcomes were therefore restricted to draw values between zero and 100. Rotarod data provided by Wang et al.^42^ was reported as a continuous variable with a minimum logical value of zero; data simulations were therefore restricted to provide positive values and no upper limit was specified. To determine whether data provided by independent experiments were compatible for meta-analysis, simulated data from the respective control groups were graphed (15,000 simulations for Demyanenko et al.,^18,19^, and 20,000 simulations for Wang et al.^42^ in order to accurately approximate the original datasets). The experiments were considered comparable if the means and standard errors of the control groups overlapped for the duration of the experimental period. Rotarod and body tilt data provided by Wang et al.^42^ fulfilled this criterion enabling direct comparison of the treatment groups from different experiments (controls from each experiment were merged to provide a common reference group). Outcomes were modelled using linear regression (rotarod, continuous data), or beta regression (body tilt, proportional data bounded from 0 to 1), as appropriate (20,000 separate models generated for each outcome assessed).^43^ Time was included as a factorial variable to enable direct comparison between groups for each assessed timepoint. Differences in the performance of the control groups presented by Demyanenko et al.,^18,19^ prevented meta-analysis of forelimb function recovery data. These data were therefore modelled separately (beta regression, 15,000 separate models) to provide a longitudinal estimate of the difference in the rate forelimb recovery between groups receiving *HDAC6* inhibitors, or vehicle (evidenced by the interaction of group and time). All presented models conformed to respective underlying assumptions based on visual inspection of diagnostics from 15 randomly selected models. Reported data detail the coefficients and 95% confidence intervals for each variable averaged across each of the simulated models. Median *p*-values, and the proportion of models in which the assessed parameter shows statistical significance are reported.

## Results

### Literature search

Searches of the Emcare (OVID), Scopus, Web of Science and Medline (OVID) databases identified a total 2446 potentially eligible articles (Figure 1). Three studies could not be retrieved as full text versions could not be found using the presented citation details. Reference list searches identified an additional study which was previously excluded as the abstract did not specifically mention HDAC6, however presented data suggested that the reported outcomes were relevant to the current review and the full text for this article was therefore screened.^44^ The full text of 68 articles were screened, of which 61 were excluded most commonly because they did not investigate the effect of *HDAC6* inhibition on AIS outcomes.

### Description of included studies

The details of the included studies are summarised in Table 1. All utilised laboratory mice (4 studies),^18,19,44,45^ or rats (3 studies),^24,42,46^ and induced AIS through photothrombosis (PTI),^18,19^ or middle cerebral artery occlusion (MCAO).^24,42,44–46^ Five studies employed commercially available *HDAC* inhibitors,^18,19,42,44,45^ of which Tubastatin A was the most widely used. Guo et al.^24^ and Han et al.^46^ investigated novel laboratory – synthesised *HDAC6* inhibitors. Several studies included groups receiving drugs targeting other HDACs,^19^ or the traditional medicine butylphthalide, for which the mechanism of action is unknown as additional comparators.^24,46,47^ For the purposes of this review, data from experimental groups receiving interventions which did not specifically target HDAC6 were omitted. Six of the seven included studies assessed the impact of administering *HDAC6* inhibitors during the acute-phase of AIS.^18,19, 24,42,46^ Follow-up for these studies was typically short (<2 weeks), with the exception of Yang et al.^44^ who reported outcomes 1 month post-AIS. Sheu et al.^45^ were the only investigators to assess the effect of administering *HDAC6* inhibitors during the sub-acute phase. In this study mice undergoing a simulated rehabilitation program received *HDAC6* inhibitors or vehicle for 2 weeks, commencing 7 days after AIS induction.

**Table 1.** Details of studies included in the systematic review.

Study	Model details		Interventions (dose and route of administration)		Drug administration	Method to assess *HDAC* inhibition *in vivo*	Stroke pathological outcomes assessed
	Organism used	Method of AIS induction	*HDAC6* inhibitor	Control intervention			
Models of permanent cerebral ischemia							
Demyanenko et al.^18^ (high ROB)	Male CD-1 mice aged 14–15 weeks	PTI	Tubastatin A (25 mg/kg i.p.)	Vehicle (5% DMSO, 2% Tween-20 in saline i.p.)	24 h post-stroke and then once/day for 2 days	Western blot assessing acetyl alpha tubulin abundance in peri-infarct tissue	• Infarct volume 4 and 7 days post-AIS (TTC stain) • Neurological function (cylinder test) 4, 7 and 14 days post-event
Demyanenko et al.^19^ (high ROB)	Male CD-1 mice aged 14–15 weeks	PTI	HPOB (10 mg/kg i.p.)	Vehicle (DMSO in saline i.p.)	1 h post-AIS, then once/day	Western blot assessing acetyl alpha tubulin abundance in peri-infarct tissue	• Infarct volume 7 days post AIS (TTC stain) • Neurological function (cylinder test and foot fault test), 4, 7 and 14 days post-AIS
Models of temporary cerebral ischaemia							
Wang et al.^42^ (medium ROB)	Male Sprague Dawley rats 240 ± 20 g	MCAO (duration of ischaemia not reported)	*Experiment 1* • Tubastatin A (25 mg/kg i.p.) • Tubastatin A (40 mg/kg i.p.) Experiment 2 • Tubastatin A (25 mg/kg i.p.)	Vehicle (5% DMSO, 2% Tween-20 in 0.9% saline i.p.)	*Experiment 1:* At reperfusion then once/day for 3 days *Experiment 2:* 24 h post-reperfusion then once/day til 3 days post-AIS	Western blot for acetyl tubulin expression in cortex and striatum in mice receiving 25 mg/kg TubA relative to sham-operated controls, 1 and 3 days post-stroke. (Timing of TubA admin relative to AIS for this assessment is unclear)	*Experiments 1 and 2:* • Infarct volume 3 days post-AIS (TTC stain) • Neurological function (rotarod performance, neurological score and body tilt percentage) daily for 3 days post-AIS
Guo et al.^24^ (medium ROB)	Male Sprague Dawley rats (age/weight not reported)	MCAO (1.5 h)	• Tubastatin A (25 mg/kg i.p.) • Compound 5 (25 mg/kg i.p.) • Compound 18 (25 mg/kg i.p.)	Unclear	Unclear (assume 1 dose upon reperfusion)	None*	• Infarct volume (TTC stain) 24 h post-AIS • Neurological deficit (neuroscore) 24 h post-AIS
Yang et al.^44^ (low ROB)	Male Thy1-YFP mice aged 8–10 weeks (20–25 g). Sex not specified	MCAO (2 h)	• Tubastatin A (25 mg/kg i.p.)	Vehicle (1% DMSO i.p.)	Upon reperfusion (number of doses unclear but assume 1)	Immunofluorescence microscopy assessing abundance of acetyl alpha tubulin	• Neurological function (pellet test, irregular ladder walking, rotarod test and cylinder test), 1 day, and weekly for 4 weeks post-AIS
Han et al.^46^ (medium ROB)	Male Sprague Dawley rats 240 ± 20 g	MCAO (1.5 h)	• Compound 3 (i.v.)	Vehicle (5% DMSO, 40% PEG, 5% Tween-80 in saline, oral gavage)	Unclear (assume 1 dose upon reperfusion)	None*	• Infarct volume 24 h post-stroke (TTC stain)
Sheu et al.^45^ (medium ROB)	Male and female C57 black J mice, 6–10 weeks old^Ϯ^	MCAO (30 or 60 min)^α^	• ACY-737 (5 mg/kg i.p.) in addition to a simulated rehabilitation programme	Vehicle (0.9% saline i.p.) in addition to a simulated rehabilitation programme	Every 2 days between 7 and 21 days post-AIS	Western blot assessing acetyl alpha tubulin abundance in the granular layer of the dentate gyrus	• Neurological function (adhesive removal test, object location test, rotarod performance) weekly for 4 weeks post-AIS

PTI: photothrombosis; i.p: intra-peritoneal; i.v: intra venous; TTC: 2,3,5-triphenyltetrazolium chloride; HPOB: 4-[(hydroxyamino)carbonyl]-N-(2-hydroxyethyl)-N-phenyl-benzeneacetamide; MCAO: middle cerebral artery occlusion – times reported relate to duration of cerebral ischaemia.

* Provided evidence of HDAC inhibition via *in vitro* assay.

Ϯ Ratio of male:female mice not reported.

α Duration of cerebral ischaemia experienced in animals assessed for neurological function unclear. Studies by Guo et al.^24^ and and Han et al.^46^ included groups of animals receiving butylphthalide as positive controls. These data were not included in the current review as the mode of action of butylphthalide is unknown and potential benefits or deficits cannot therefore be attributed to HDAC6 inhibition.^47^ Outcomes of risk of bias (ROB) assessments for each study are summarised in the first column.

### Risk of bias assessment

Three included studies were identified to be at high risk of bias, three of medium risk of bias and one of low risk of bias (Table 1, Supplement 2). Three studies described the AIS induction methodologies in sufficient detail to permit direct replication,^24,44,45^ and two^44,46^ reported using measures to ensure that AIS induction procedures were successful. All studies but one,^46^ provided data demonstrating that the administered drugs successfully inhibited HDAC6 *in vivo*, four clearly described the drug administration regime (e.g. timing, and frequency of administration).^18,19,42,45^ Three studies reported randomising rodents to experimental groups,^42,44,46^ two utilised outcome assessors blinded to group allocation,^24,44^ and one provided a power calculation to justify sample sizes.^46^ No study referenced the STAIR preclinical research recommendations,^36,37^ or utilised rodent models incorporating common comorbidities or clinical risk factors for AIS. All studies but one,^45^ used male animals exclusively.

### Outcome assessments

The difference in the size of the cerebral infarctions developed by animals receiving *HDAC6* inhibitors or controls evidenced by TTC staining was the most commonly assessed outcome.^18,19,24,42,46^ Neurological outcomes were less frequently reported, and significant differences in the methods used to assess functional recovery was observed (Tables 1 and 2). Tests of forelimb function (cylinder test),^18,19,44^ and the ability to balance on an accelerating rotating rod (rotarod),^42,44,46^ were each employed in three studies. Other approaches to assess neurological function included tests of locomotory performance, body tilting, the ability to locate or retrieve an object, or remove adhesive labels, and a composite neurological deficit score were less consistently applied.

### The impact of *HDAC6* inhibition on cerebral infarction size

**Figure 2.**
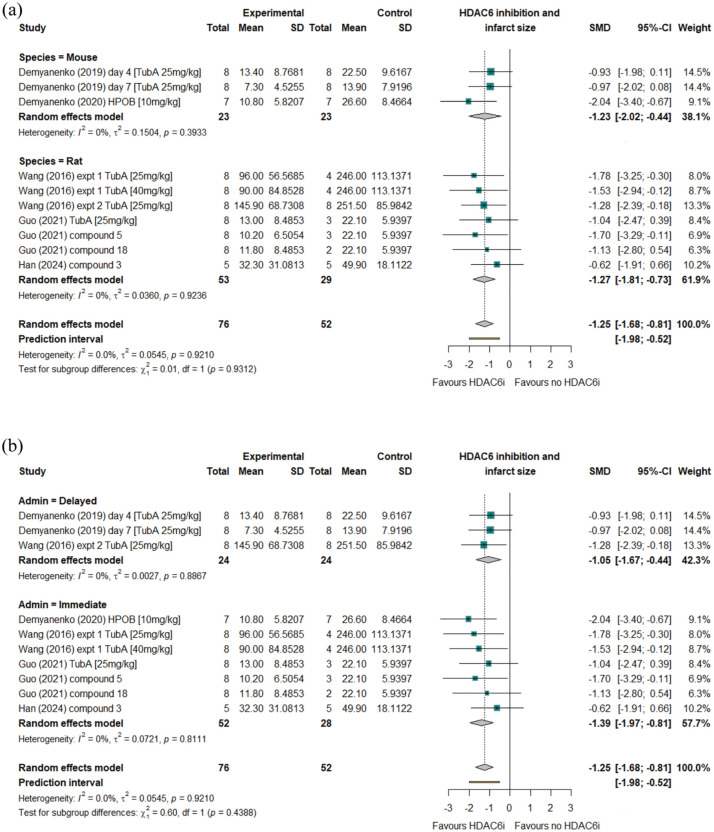
Forest plots showing outcomes of random effects analysis of data from studies reporting the effects of *HDAC6* inhibition on cerebral infarction volume. Findings are subset by rodent species (a) or timing of *HDAC6* inhibitor administration relative to AIS induction (b).

The difference in cerebral infarction size between groups of animals receiving *HDAC6* inhibitors or control interventions was reported in five studies which collectively investigated seven different *HDAC6* inhibitors or doses in total of 128 animals (Table 1, Figure 2).^18,19,24,42,46^ The timing of infarct size assessment relative to AIS induction varied between studies ranging from 24 h to 7 days post-AIS. All reported a trend towards smaller cerebral infarctions in animals receiving *HDAC6* inhibitors than controls, however this did not reach statistical significance in all studies (Table 2). Meta-analysis including all studies highlighted a strong effect of *HDAC6* inhibition in reducing cerebral infarction size (SMD: −1.25; 95% CI: −1.68, −0.81; Figure 2). No significant inter-study heterogeneity was observed (*I*^2^ = 0%) and no publication bias was evident (Supplement 4). Trim and fill analyses suggested an additional two studies were needed to provide perfect symmetry in the Funnel plots. Imputing data that these studies would be predicted to provide did not markedly alter findings (standard mean difference: −1.11; 95% CI: −1.53, −0.68), *p* < 0.0001, Supplement 4). Leave-one-out sensitivity analyses suggested that the effect of *HDAC6* inhibition on infarction size was not dependent on data from any single study (Supplement 4). Sub-analysis highlighted that the strength of the effect of *HDAC6* inhibition on infarction volume was similar between species (SMD: −1.23; 95% CI: −2.02, −0.44 and SMD: −1.27; 95% CI: −1.81, −0.73; for mice or rats respectively, *p* = 0.931; Figure 2(a)) and between those receiving HDAC6 inhibitors rapidly, or after a delay (SMD: −1.39; 95% CI: −1.9, −0.81 and SMD: −1.05; 95% CI: −1.67, −0.44; respectively, *p* = 0.439; Figure 2(b)). Meta-regression demonstrated that the difference in infarction size between groups was not influenced by the timing of tissue assessment (days post-AIS), or the number of doses administered (Supplement 7). The association of *HDAC6* inhibition with smaller cerebral infarction sizes was upheld in sensitivity analyses incorporating data from studies utilising (i) commercially available *HDAC6* inhibitors and (ii) the most widely employed *HDAC6* inhibitor (Tubastatin A, 25 mg/kg, Supplements 5 and 6).

**Table 2.** Summary of reported outcomes from independent studies.

Study/group	Outcome assessments								
	Cerebral infarction size	Neurological deficit score	Cylinder test	Foot fault test	Rotarod performance	Body tilt	Adhesive removal	Object location test	Pellet retrieval
Demyanenko et al.^18^	↓*		↑	↔^α^					
Demyanenko et al.^19^	↓		↑						
Sheu et al.^45^					↔		↓	↓	
Yang et al.^44^			↑	↑^α^	↑				↑
Han et al.^46^	↓								
Wang et al.^42^									
Tubastatin 25 mg/kg (rapid administration)	↓	↓			↑	↑			
Tubastatin 40 mg/kg (rapid administration)	↓	↓			↔	↑			
Tubastatin 25 mg/kg (delayed administration (24 h post-AIS))	↓	↓			↑	↔			
Guo et al.^24^									
Tubastatin A, 25 mg/kg	↔	↔							
Compound 5, 25 mg/kg	↓	↓							
Compound 18, 25 mg/kg	↔	↔							

Detailing the outcomes observed for the animals receiving *HDAC6* inhibitors relative to controls (as detailed in Table 1). Where appropriate outcomes of investigations involving multiple *HDAC6* inhibitors, or modes of administration are separately reported. Downwards arrows for cerebral infarction size and neurological deficit score indicate that these outcomes were reported to be less severe for animals receiving *HDAC6* inhibitors. For all other tests, upwards and downwards arrows denote statistically significantly improved or poorer performance (respectively) in animals receiving *HDAC6* inhibitors than controls. Sidewards arrows indicate no significant difference in performance between groups. Blank cells indicate that the outcome was not assessed by the study authors.

* Authors assessed brains collected at days 4 and 7 post-AIS and reported that animals receiving *HDAC6* inhibitors had significantly smaller infarctions than controls at both timepoints.

α Different approaches were used to assess this outcome (Demyanenko et al.^18^: grid walking, Yang et al.^44^: irregular ladder walking). These have been grouped for simplicity in the current article.

### The influence of *HDAC6* inhibition on the recovery of forelimb function

**Figure 3.**
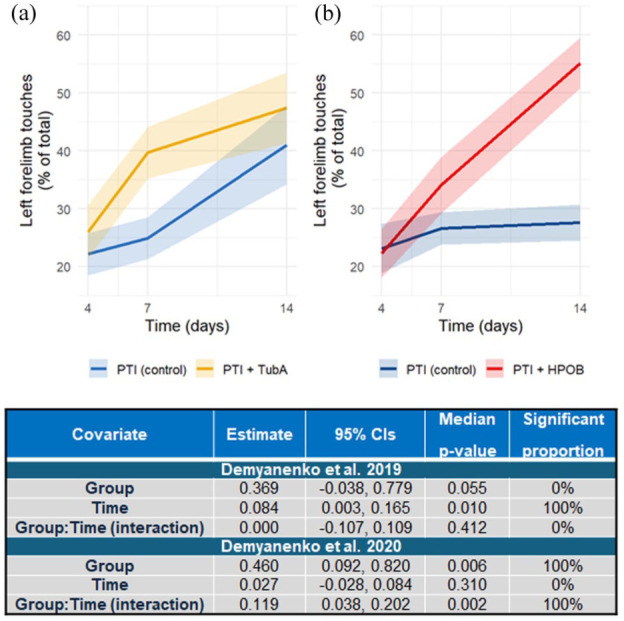
Plots showing outcomes from data simulations detailing recovery of left forelimb function in mice receiving *HDAC6* inhibitors or controls based on data reported by Demyanenko et al.^18,19^ in their 2019 (a), or 2020 (b) papers. Employed *HDAC6* inhibitors were Tubastatin A (25 mg/kg; Demyanenko et al.^18^) or HPOB (10 mg/kg; Demyanenko et al.^19^). Controls in both studies were mice that had undergone PTI and were receiving vehicle. Data are reported as means (solid lines) and standard errors (halos) as in the original studies. Inset tables detail the results of intergroup comparisons for each timepoint following modelling analysis of the 15,000 simulated datasets. Reported data detail the estimates for each covariate and covariate interaction (averaged across 15,000 models) and the median p-value across all models. Significant proportion refers to the percentage of the 15,000 models that each comparison showed statistical significance.

Three studies presented data reporting the recovery of left forelimb function post-AIS. Demyanenko et al.^18,19^ detailed the recovery of left forelimb function (reported as the percent of total contacts with the wall of a glass cylinder made with the left forelimb over 3 min at 1, 7 and 14 days post-AIS) in animals receiving either Tubastatin A (25 mg/kg) or HPOB (: 4-[(Hydroxyamino)carbonyl]-N-(2-hydroxyethyl)-N-phenyl-benzeneacetamide, 10 mg/kg). Reported data suggested that animals receiving Tubastatin A showed significantly greater left forelimb use than controls 7 days post-AIS, but not at any other time,^18^ whereas those receiving HPOB showed greater recovery of forelimb function after 14 days.^19^ Data modelling demonstrated that mice receiving HPOB (10 mg/kg) recovered left forelimb function more rapidly than vehicle controls (exhibiting an increase of left forelimb touches of 0.119 (95% CI: 0.038, 2.02) percent/day compared to controls, *p* = 0.002). Those receiving Tubastatin A (25 mg/kg) demonstrated a daily increase in left forelimb touches of 0.0 (95% CI: −0.107, 0.109), percent/day which did not differ significantly from vehicle controls (*p* = 0.412, Figure 3). Yang et al.^44^ reported that the recovery of forelimb function in the 4 weeks following AIS was significantly greater in animals receiving Tubastatin A (25 mg/kg) compared to vehicle controls however data could not be extracted from presented graphs, prohibiting reanalysis.

### The influence of HDAC6 inhibition on rotarod performance

**Figure 4.**
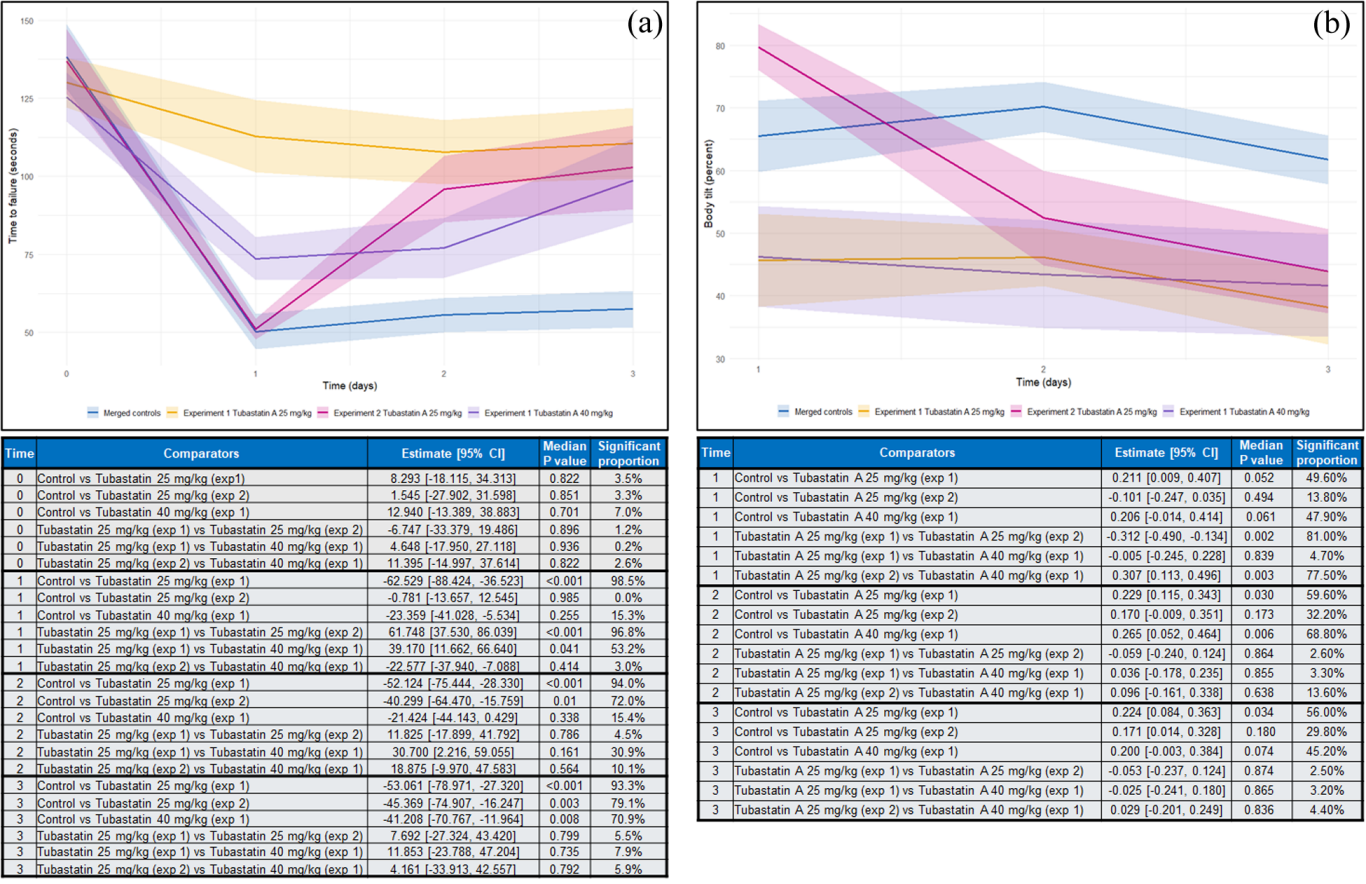
Plots showing outcomes from data simulations detailing rotarod performance (a) and body tilting (b) in animals receiving *HDAC6* inhibitors or controls based on data reported by Wang et al.^42^ Graphs show merged data from two compatible experiments for each outcome. Experiment 1 included animals receiving Tubastatin A (25 mg/kg) immediately upon reperfusion following MCAO. Experiment 2 included animals receiving Tubastatin A (25 mg/kg) 24 h post-MCAO. Data from the control groups in each experiment were merged for the purposes of this analysis. Data are shown as means (solid lines) and standard errors (halos) as in the original studies. Inset tables detail the results of intergroup comparisons for each timepoint following modelling analysis of the 20,000 simulated datasets. Reported data detail the estimated inter-group differences (averaged across 20,000 models) and the median p-value across all models. Significant proportion refers to the percentage of the 20,000 models that each comparison showed statistical significance.

Wang et al.,^42^ Sheu et al.^45^ and Yang et al.^44^ presented data detailing the post-AIS rotarod performance of animals receiving HDAC6 inhibitors or vehicle. Wang et al.^42^ reported that rotarod performance of animals receiving 25 mg/kg Tubastatin A at the time of reperfusion was significantly better than vehicle controls, whereas those receiving 40 mg/kg Tubastatin A exhibited similar performance to the control group. In a second experiment, rats receiving Tubastatin A (25 mg/kg) 24 h after MCAO exhibited significantly better rotarod performance when assessed 3 days post-AIS than the control group, despite similar deficits prior to drug administration. Data modelling revealed that by day three, there was no significant difference in the rotarod performance of animals receiving Tubastatin A at any dose and all animals receiving Tubastatin A performed significantly better than vehicle controls (Figure 4).

Yang et al.^44^ also reported that animals receiving Tubastatin A (25 mg/kg) showed greater improvements in rotarod performance than vehicle controls in the 4 weeks following AIS induction, however this was only demonstrated to be statistically significant different from 2 weeks post-AIS. In contrast, Sheu et al.^45^ reported no difference in the rotarod performance of mice receiving HDAC6 inhibitors or vehicle. Data presented by Yang et al.^44^ or Sheu et al.^45^ could not be accurately extracted, preventing longitudinal modelling of these outcomes.

### The influence of *HDAC6* inhibition on neurological deficit following AIS

Two studies investigated whether *HDAC6* inhibition influenced the degree of neurological deficit post-AIS.^24,42^ Wang et al.^42^ assessed neurological function using a 7-point scoring system based on the outcomes from a battery of motor, sensory and reflex tests over the 3 days post-AIS. Reported neurological function in animals receiving either 25 or 40 mg/kg Tubastatin A at the time of reperfusion was significantly better than those receiving vehicle at days 1–3, with no statistically significant difference in the neurological scores of animals receiving Tubastatin A at 25 or 40 mg/kg.^42^ A second experiment demonstrated that rats receiving Tubastatin A (25 mg/kg) 24 h post-AIS experienced significantly improved neurological function 3 days post-AIS than vehicle controls, despite similar initial symptom severity. Presented data suggest that those receiving the delayed treatment had a higher neurological deficit score than those receiving 25 mg/kg Tubastatin A at the time of reperfusion (mean (+SEM) neurological scores 2.1 + 0.4 vs 4.1 + 0.5, respectively), however, data could not be formally meta-analysed and compared.^42^ Contrasting data were provided by Guo et al.^24^ who reported that animals receiving the novel drug ‘compound 5’, but no other HDAC6 inhibitor (including 25 mg/kg Tubastatin A) exhibited a statistically significant reduction in neurological deficit compared to vehicle controls, 24 h-post AIS.

### The impact of *HDAC6* inhibition on locomotory function post-AIS

Demyanenko et al.^18^ reported that animals receiving 25 mg/kg Tubastatin A performed marginally better on a grid walking test than vehicle controls 4 and 7 days post-AIS, whereas performance at 14 days was similar between the groups. No significant difference in grid walking performance between groups receiving Tubastatin A or vehicle was observed when data were analysed longitudinally (*p*-trend = 0.058). Yang et al.^44^ utilised a similar irregular ladder test and reported that mice receiving Tubastatin A (25 mg/kg) exhibited significantly fewer foot faults (slips) than those receiving vehicle from 1 to 4 weeks post-AIS, despite similar performance 24 h following AIS induction. Meta-analysis was not possible for these outcomes.

### The impact of HDAC6 on other functional outcomes

Reported findings from experiments investigating less frequently assessed neurological outcomes are summarised in Table 2. Wang et al.^42^ reported that rats receiving Tubastatin A (25 or 40 mg/kg) immediately after MCAO (experiment 1), exhibited a significantly less pronounced body tilt than those receiving vehicle. They further reported that the body tilt percentage those receiving Tubastatin A (25 mg/kg) 24 h post-AIS (experiment 2) was no different to the control groups. Meta-analysis of these data confirmed that the body tilt percentage of mice receiving Tubastatin A 24 h post-AIS was significantly worse than those receiving the drug at reperfusion, at all assessed timepoints (Figure 4(b)).

Yang et al.^44^ demonstrated that mice receiving Tubastatin A (25 mg/kg) exhibited greater ability to retrieve a food pellet than vehicle controls 1–4 weeks after AIS induction, despite similar performances from each group 1 day post-MCAO. Conversely Sheu et al.^45^ reported that mice receiving *HDAC6* inhibitors as an adjunct to physical rehabilitation performed significantly worse at adhesive removal and object location tests than those receiving vehicle (plus rehabilitation).

## Discussion

Findings from the current meta-analysis indicate that pharmacologically inhibiting *HDAC6* during the hyperacute phases of AIS confers significant protection, evidenced by smaller infarction sizes and greater recovery of motor function, compared to controls. Data suggest functional outcomes can be significantly improved if *HDAC6* inhibition is instigated up to 24 h post-event suggesting that this strategy may provide particular benefit for late-presenting patients, or those who require transportation for specialist assessment. Evidence suggests that *HDAC6* inhibition during the sub-acute phase of AIS may prove to be harmful indicating a nuanced role for *HDAC6* in the response to cerebral ischaemia.^45^

The mechanisms by which *HDAC6* inhibition reduces the severity of AIS-induced damage and neurological impairment remains unclear. The major non-histone substrate for *HDAC6* in the brain is alpha-tubulin,^27^ which when acetylated, plays a structural role in neuronal microtubules and dendritic spines, thereby maintaining connections between adjacent cells.^24,48^ Evidence suggests that *HDAC6* plays a key role in de-acetylating alpha tubulin following AIS which destabilises microtubule structure, leading to impaired neuronal function and decreased capacity for subsequent synaptogenesis.^42^ The preservation of endogenous acetyl alpha tubulin within the brain is therefore suggested to at least in part explain the observed protection afforded by *HDAC6* inhibition.^18,19,24,42,46,44^ This, however, may be time- and context-dependent as Sheu et al.^45^ argue that the poorer performance of animals receiving *HDAC6* inhibitors in the week following AIS was due to an over-abundance of alpha tubulin which impaired the maturation of newly formed neurons. Guzenko et al.^26^ further reported that inhibiting *HDAC6* with Tubastatin A prevented the deacetylation of the protein p53 at lysine 320 following AIS induction, which in turn significantly reduced the extent of p53-mediated apoptosis in the peri-infarct tissues compared to controls. Others have also reported that *HDAC6* inhibition reduces the severity of neuro-inflammation following AIS.^49,50^ Thus, available evidence suggests that the effects of inhibiting *HDAC6* in the hyper acute and acute phases of AIS is multifaceted. Unbiased analyses using modern genomic and proteomic approaches to determine the pathways and cells influenced by *HDAC6* inhibition are required.

There is, as yet, no evidence from Randomised Controlled Trials to support or refute the safety or practicality of an AIS management strategy based on *HDAC* inhibition. Findings from the current SOLVE study, a phase two randomised placebo-controlled trial testing the ability for sodium valproate to improve 90 day outcomes for patients experiencing AIS will provide valuable insight to the potential utility of pan HDAC inhibition in this patient population (https://clinicaltrials.gov/study/NCT06020898 accessed August 2025).^23^ It is important to note, however, that sodium valproate does not target *HDAC6* and this trial will therefore not directly assess whether *HDAC6* inhibition benefit patients experiencing AIS. Clinical trials assessing the benefits of the specific *HDAC6* inhibitor ACY-1215 (ricolinostat) for other indications report that the drug is well tolerated, supporting the notion that employing selective *HDAC6* inhibitors may bypass the adverse effects associated with pan-HDAC inhibition.^51,52^ The chemical structure of ricolinostat has been suggested to limit bioavailability in the brain which limits its utility in AIS management.^24^ Tubastatin A was the most commonly investigated HDAC6 inhibitor however the uptake of Tubastatin A by the brain is typically low owing to the presence of a constituent hydroxamate moiety, which potentially limits the practical value of this drug. Despite this, the included studies were able to demonstrate acetyl-alpha tubulin preservation in the brains of animals receiving Tubastatin A, suggesting that the drug is able to reach the site of pathology in therapeutic doses following AIS. It is possible that the characteristic increase in blood-brain-barrier following AIS facilitates the entry of the Tubastatin A into the brain, however there is interest in developing highly brain penetrant *HDAC6* inhibitors which do not include the hydroxamate group and may have improved bioavailability.^24,34,46,53,54^ For example, the studies by Guo et al.^24^ (2021) and Han et al.^46^ included in the current review detailed outcomes of experiments which sought to develop highly brain permeable experimental *HDAC6* inhibitors. Of note, Guo et al.^24^ reported that their novel tetrahydrobenzazepine-containing *HDAC6* inhibitor ‘Compound 5’ rapidly accumulated at high concentration, and meta-regressions presented here demonstrate that a single dose of this compound produced a greater reduction in infarction volume than comparable doses of Tubastatin A. It is further suggested that the development of alternative *HDAC6* inhibitors may alleviate safety concerns associated with hydroxamate-containing agents due to their mutagenic potential.^55^ None of the included studies have reported safety outcomes for animals receiving Tubastatin A, and no registered clinical trials investigating this drug for any indication could be found (www.clinicaltrials.gov, www.anzctr.org.au accessed August 2025), meaning that the acceptability or utility of a Tubastatin A-based therapy for AIS remains unclear. Emerging data however suggest that the development of next-generation *HDAC6* inhibitors may deliver new drugs with greater potential to safely improve patient outcomes.

The presented analyses must be considered in light of the inherent strengths and weaknesses. Significant emphasis was placed on the assessment of neurological outcomes as recommended by the STAIR consortium,^36,37^ rather than focusing solely on infarction size as seen in previous analyses.^21^ Moreover, the employed modelling approaches enabled us to combine individually presented longitudinal datasets and statistically compare the assess the influence of different doses of *HDAC6* inhibitors, and timing of administration, on the several functional outcomes. It is notable that our systematic literature search identified only seven articles investigating the potential for HDAC6 inhibition to improve AIS outcomes and only one identified study was suggested to have a low risk of overall bias.^44^ Importantly, meta-analysis assessing the influence of *HDAC6* inhibition on cerebral infarction volume did not suggest any publication bias. However, key gaps in current data including a lack of female representation, use of young, healthy animals and no assessment of adverse reactions to any of the tested agents or their compatibility with pharmacological reperfusion agents as suggested in the recent STAIR guidelines,^36,37^ complicates translation of potentially encouraging findings from rodent models. Moreover, most identified studies assessed therapeutic efficacy by measuring cerebral infarction size, whereas the impact of *HDAC6* inhibition on neurological function was less widely reported and was assessed using varied tests which could not be easily compared. Thus, the potential for *HDAC6* inhibition to improve functional outcomes requires further investigation, especially given the evidence provided by Sheu et al.^45^ which suggests that modulating *HDAC6* activity during the sub-acute phase may be potentially detrimental. Similarly, none of the included studies investigated whether *HDAC6* inhibition provided protection against other aspects of AIS pathophysiology, such as blood-brain-barrier destabilisation,^34^ meaning that the understanding of the mechanisms underpinning the apparent benefits of *HDAC6* inhibition is incomplete. Finally, the current analysis does not provide insight into whether the protective effects of *HDAC6* inhibition may be enhanced if used in combination with agents targeting other HDAC family members. The studies by Guo et al.^24^ and Han et al.^46^ both included a group of animals receiving butylphthalide, a non-specific traditional medication) as positive controls, and presented data tentatively indicate that this group experience conferred greater reductions in cerebral infarction volume than those receiving specific *HDAC6* inhibitors. In contrast, Demyanenko et al.^18^ reported that infarction sizes were similar between groups of animals receiving HPOB, or sodium valproate highlighting the need for additional studies in this area. Importantly, Tubastatin A has also been demonstrated to inhibit *HDAC10* which belongs to the same enzyme class as *HDAC6*.^56,57^ None of the studies included in the current review which employed Tubastatin A assessed the concomitant of the drug on *HDAC10* activity. Moreover the recent review by Madji et al.^21^ did not identify any studies which directly assessed the influence of *HDAC10* on AIS outcomes. Thus the possibility that the reported beneficial effects of Tubastatin A on AIS recovery are a consequence of inhibiting both HDAC6 and 10 cannot be discounted. Future studies investigating the specific contributions of HDACs 6 and 10 to AIS pathobiology are therefore warranted.

In conclusion, available data suggests considerable potential for *HDAC6* inhibition to improve outcomes following AIS, evidenced by reductions in the severity of brain infarction and improvements in neurological function, even when treatment is delayed by up to 24 h. Data position the well characterised compound Tubastatin A as a lead candidate for future investigation, however the development of new drugs with high ability to cross the blood brain barrier may yield new agents with greater cytoprotective potential. Additional experiments which (i) more closely simulate the clinical scenario, (ii) investigate the longer-term impacts of *HDAC6* inhibition on AIS outcomes and (iii) utilise unbiased screening approaches to elucidate the mechanisms by which *HDAC6* inhibition protects against AIS-induced damage are needed to progress this promising avenue.

## Supplemental Material

sj-pdf-1-jcb-10.1177_0271678X251405674 – Supplemental material for A systematic review and meta-analysis assessing the influence of histone deacetylase 6 inhibition on brain infarction and neurological function following acute ischaemic stroke in rodent models Supplemental material, sj-pdf-1-jcb-10.1177_0271678X251405674 for A systematic review and meta-analysis assessing the influence of histone deacetylase 6 inhibition on brain infarction and neurological function following acute ischaemic stroke in rodent models by Oliver B Ma, Timothy C Noack, Alexandra F. Trollope and Joseph V Moxon in Journal of Cerebral Blood Flow & Metabolism
