# Supplementary material for: A systematic review and meta-analysis assessing the influence of histone deacetylase 6 inhibition on brain infarction and neurological function following acute ischaemic stroke in rodent models
Source: J Cereb Blood Flow Metab. 2026 Jan 18:0271678X251405674. Online ahead of print. doi: 10.1177/0271678X251405674 (PMC12815625; doi:10.1177/0271678X251405674)
Supplement: sj-pdf-1-jcb-10.1177_0271678X251405674 – Supplemental material for A systematic review and meta-analysis assessing the influence of histone deacetylase 6 inhibition on brain infarction and neurological function following acute ischaemic stroke in rodent models [file sj-pdf-1-jcb-10.1177_0271678X251405674.pdf]

A systematic review and meta-analysis assessing the influence of histone deacetylase 6 inhibition on brain infarction and neurological function following acute ischaemic stroke in rodent models

Oliver B. Ma, Timothy C. Noack, Alexandra F. Trollope, Joseph V. Moxon

Supplementary material

Supplement 1: Literature search protocol

Supplement 2: Risk of bias assessment for included studies

Supplement 3: Data used to generate random effects meta-analysis comparing cerebral infarction size in animals receiving HDAC6 inhibitors or control interventions:

A) Cerebral infarction data used for meta-analysis using data from all studies

B) Cerebral infarction data used for meta-analysis using data from studies using commercially available HDAC6 inhibitors

C) Cerebral infarction data used for meta-analysis from studies using Tubastatin A (25 mg/kg).

Supplement 4: Funnel plots, trim and fill analyses and leave one sensitivity analysis of infarct data considering data from all studies.

Supplement 5. Forest plots detailing impact of HDAC6 inhibition on cerebral infarction size when considering data from experiments using commercially available HDAC6 inhibitors.

Supplement 6. Forest plots detailing impact of HDAC6 inhibition on cerebral infarction size when considering data from experiments using 25 mg/kg Tubastatin A to inhibit HDAC6.

Supplement 7: Outcomes of meta-regression assessing the relationship of the time of tissue harvest (A) and number of drug doses administered (B) with the difference in cerebral infarction size between animals receiving HDAC6 inhibitors or control interventions.

Supplement 8: Modelling the recovery of left forelimb function as reported by Demyanenko *et al.* (2019 and 2020):

A) Reported vs simulated data for Demyanenko cylinder test analyses.

B) Comparing left forelimb recovery in control groups reported by Demyanenko 2019 and 2020.

Supplement 9: Reported vs simulated data for functional outcomes reported by Wang *et al.* (2016):

A) Comparing longitudinal outcomes between control groups in separate experiments reported by Wang *et al.* (2016).

Supplement 1: Literature search protocol

The MEDLINE (via OVID), EMCARE (via OVID), SCOPUS and Web of Science databases were searched with the assistance of a librarian who specialised in medical database searches. MEDLINE (via OVID) and EMCARE (via OVID) were searched through the use of MeSH search terms: “HDAC6 inhibition” OR “HDAC6” OR “histone deacetylase 6” AND “stroke”. These databases’ search results were used to create the following search string which was then used to search SCOPUS and Web of Science:

"HDAC6 inhibition" OR "ec 3.5.1.98" OR "histone deacetylase 6" OR "HDAC6 protein" OR "protein HDAC6" OR "class i histone deacetylases" OR "class ii histone deacetylases" OR "hdac proteins" OR "histone deacetylase" OR "histone deacetylase complexes" OR "histone deacetylases" OR "histone deacetylase inhibitor" OR "histone deacetylase inhibitors" OR "histone deacetylation" OR "HDAC6" OR "HDAC6 protein" OR "protein HDAC6" OR "dominostat" OR "4sc 202" OR "HDAC1" OR "HDAC1 protein" OR "protein HDAC1" OR "HDAC10" OR "HDAC10 protein" OR "protein HDAC10" OR "HDAC11" OR "HDAC11 protein" OR "protein HDAC11" OR "HDAC2" OR "HDAC2 protein" OR "protein HDAC2" OR "group III histone deacetylases" OR "HDAC3" OR "HDAC3 protein" OR "protein HDAC3" OR "HDAC4" OR "HDAC4 protein" OR "protein HDAC4" OR "antigen NY CO 9" OR "HDAC5" OR "HDAC5 protein" OR "NY CO 9 antigen" OR "NY CO 9 protein" OR "protein HDAC5" OR "protein NY CO 9" OR "HDAC7" OR "HDAC7 protein" OR "protein HDAC7" OR "HDAC8" OR "HDAC8 protein" OR "protein HDAC8" OR "HDAC9" OR "HDAC9 protein" OR "protein HDAC9" OR "histone deacetylase 1" OR "histone deacetylase 2" OR "histone deacetylase 3" OR "histone deacetylase 4" OR "histone deacetylase 5" OR "histone deacetylase 6" OR "histone deacetylase 7" OR "histone deacetylase 8" OR "histone deacetylase 9" OR "histone deacetylase 10" OR "histone deacetylase 11" OR "HDAC1 gene" OR "HDAC2 gene" OR "HDAC3 gene" OR "HDAC4 gene" OR "HDAC5 gene" OR "HDAC6 gene" OR "HDAC7 gene" OR "HDAC8 gene" OR "HDAC9 gene" OR "HDAC10 gene" OR "HDAC11 gene"

AND "brain ischemia" OR "brain ischaemia" OR "brain ischemias" OR "brain ischaemias" OR "cerebral ischemia" OR "cerebral ischaemia" OR "cerebral ischemias" OR "cerebral ischaemias" OR "ischemic encephalopathies" OR "ischaemic encephalopathies" OR "ischemic encephalopathy" OR "ischaemic encephalopathy" OR "brain infarction" OR "anterior cerebral circulation infarction" OR "anterior circulation brain infarction" OR "brain infarctions" OR "brain infarcts" OR "brain venous infarction" OR "brain venous infarctions" OR "posterior circulation brain infarction" OR "venous brain infarction" OR "venous brain infarctions" OR "benedict syndrome" OR "brain stem infarction" OR "brain stem infarctions" OR "brain stem infarcts" OR "brainstem stroke" OR "claude syndrome" OR "foville syndrome" OR "millard gublar syndrome" OR "top of the basilar syndrome" OR "weber syndrome" OR "anterior choroidal artery infarction" OR "cerebral infarction" OR "cerebral infarctions" OR "cerebral infarcts" OR "posterior choroidal artery infarction" OR "subcortical infarction" OR "subcortical infarctions" OR "ischemic stroke" OR "ischemic strokes" OR "cryptogenic embolism stroke" OR "cryptogenic embolism strokes" OR "cryptogenic ischemic stroke" OR "cryptogenic ischemic strokes" OR "cryptogenic stroke" OR "cryptogenic strokes" OR "ischaemic stroke" OR "ischaemic strokes" OR "cryptogenic ischaemic stroke" OR "cryptogenic ischaemic strokes" OR "ischemia reperfusion injury" OR "ischemia-reperfusion injuries" OR "reperfusion damage" OR "reperfusion damages" OR "reperfusion injuries" OR "reperfusion injury" OR "ischaemia reperfusion injury" OR "ischaemia-reperfusion injuries" OR "brain arterial insufficiency" OR "aca infarction" OR "aca infarctions" OR "aca infarcts" OR "anterior cerebral artery stroke" OR "anterior cerebral artery syndrome" OR "heubner artery infarction" OR "heubner's artery infarction" OR "heubners artery infarction" OR "middle cerebral artery infarction" OR "left middle cerebral artery infarction" OR "mca infarction" OR "mca infarcts" OR "middle cerebral artery circulation infarction" OR "middle cerebral artery embolic infarction" OR "middle cerebral artery embolus" OR "middle cerebral artery

occlusion" OR "middle cerebral artery stroke" OR "middle cerebral artery syndrome" OR "middle cerebral artery thrombosis" OR "middle cerebral artery thrombotic infarction" OR "right middle cerebral artery infarction" OR "pca infarction" OR "pca infarcts" OR "posterior cerebral artery embolic infarction" OR "posterior cerebral artery stroke" OR "posterior cerebral artery syndrome" OR "posterior cerebral artery thrombotic infarction" OR "cardio embolic stroke" OR "cardio-embolic strokes" OR "embolic stroke" OR "embolic strokes" OR "acute thrombotic stroke" OR "acute thrombotic strokes" OR "thrombotic stroke" OR "thrombotic strokes" OR "Ischemic stroke" OR "ischaemic stroke" OR "brain ischemia" OR "brain ischaemia" OR "cerebrovascular accident" OR "acute cerebrovascular lesion" OR "acute focal cerebral vasculopathy" OR "stroke" OR "apoplectic stroke" OR "apoplexia" OR "apoplexy" OR "blood flow disturbance, brain" OR "brain accident" OR "brain attack" OR "brain blood" OR "flow disturbance" OR "brain insult" OR "brain insultus" OR "brain vascular accident" OR "cerebral apoplexia" OR "cerebral insult" OR "cerebral stroke" OR "cerebral vascular accident" OR "cerebral vascular insufficiency" OR "cerebro vascular accident" OR "cerebrovascular arrest" OR "cerebrovascular failure" OR "cerebrovascular injury" OR "cerebrovascular insufficiency" OR "cerebrovascular insult" OR "cerebrum vascular accident" OR "cryptogenic stroke" OR "CVA" OR "insultus cerebralis" OR "ischaemic seizure" OR "ischemic seizure" OR "thrombotic stroke" OR "acute ischemic stroke" OR "acute ischaemic stroke" OR "anterior circulation ischaemic stroke" OR "anterior circulation ischemic stroke" OR "anterior circulation stroke syndrome" OR "anterior circulatory stroke" OR "anterior circulation ischemia" OR "anterior circulation ischemic event" OR "anterior circulation ischaemia" OR "anterior circulation infarction" OR "anterior circulation stroke" OR "anterior circulation brain infarct" OR "anterior circulation brain infarction" OR "anterior circulation infarct" OR "anterior circulation ischemic infarct" OR "anterior circulation ischaemic infarct" OR "anterior circulation ischemic infarction" OR "anterior circulation ischaemic infarction" OR "anterior circulatory infarct" OR "anterior circulatory infarction" OR "partial anterior circulation infarct" OR "PACI (partial anterior circulation infarct)" OR "partial anterior circulation brain infarct" OR "partial anterior circulation brain infarction" OR "partial anterior circulation infarction" OR "partial anterior circulatory infarct" OR "partial anterior circulatory infarction" OR "chronic ischemic stroke" OR "chronic ischaemic stroke" OR "cryptogenic ischemic stroke" OR "cryptogenic embolic stroke" OR "cryptogenic ischaemic stroke" OR "posterior circulation stroke" OR "posterior circulation ischaemic stroke" OR "posterior circulation ischemic stroke" OR "posterior circulation stroke syndrome" OR "posterior circulatory stroke" OR "posterior circulation ischemia" OR "posterior circulation ischaemia" OR "posterior circulation ischemic event" OR "posterior circulation ischaemic event" OR "subacute ischemic stroke" OR "sub−acute ischaemic stroke" OR "sub−acute ischemic stroke" OR "subacute ischaemic stroke" OR "wake up stroke" OR "awakening stroke" OR "stroke at awakening" OR "stroke at wake up" OR "wakeup stroke" OR "acute brain ischaemia" OR "acute brain ischemia" OR "brain arterial insufficiency" OR "brain circulation disorder" OR "cerebrovascular disease" OR "cerebral blood circulation disorder" OR "cerebral blood flow disorder" OR "cerebral circulation disorder" OR "cerebral circulatory disorder" OR "cerebrovascular circulation disorder" OR "cerebrovascular ischaemia" OR "cerebrovascular ischemia" OR "ischaemia cerebri" OR "ischaemic brain disease" OR "ischemia cerebri" OR "ischemic brain disease" OR "neural ischaemia" OR "neural ischemia" OR "experimental cerebral ischemia" OR "experimental brain ischemia" OR "experimental cerebral ischaemia" OR "experimental ischaemic stroke" OR "experimental ischemic stroke" OR "experimentally induced brain ischemia" OR "experimentally induced cerebral ischaemia" OR "experimentally induced cerebral ischemia" OR "experimentally induced ischemic stroke" OR "experimentally induced ischaemic stroke" OR "brain infarction" OR "brain cortex infarct" OR "brain cortex infarction" OR "brain infarct" OR "cerebral cortex infarct" OR "cerebral cortex infarction" OR "cerebral infarct" OR "cerebral infarction" OR "cerebrovascular infarct" OR "cerebrovascular infarction" OR "cortical infarct" OR "cortical infarction" OR "hemisphere infarct" OR "hemisphere infarction" OR "hemispheric infarct" OR "hemispheric infarction" OR "infarction, brain" OR "silent brain infarction" OR "total anterior circulation infarct" OR "TACI (total anterior circulation infarct)" OR "total anterior circulation

brain infarct" OR "total anterior circulation brain infarction" OR "total anterior circulation infarction" OR "total anterior circulatory infarct" OR "total anterior circulatory infarction" OR "cerebral ischemia reperfusion injury" OR "cerebral ischaemia reperfusion injury" OR "brain IR injury (reperfusion)" OR "brain IRI" OR "brain ischaemia reperfusion injury" OR "brain ischaemic reperfusion injury" OR "brain ischemia reperfusion injury" OR "brain ischemia/reperfusion (IR) injury" OR "brain ischemic reperfusion injury" OR "brain reperfusion injury" OR "cerebral IR injury (reperfusion)" OR "cerebral IRI" OR "cerebral ischaemia/reperfusion (IR) injury" OR "cerebral ischaemic reperfusion injury" OR "cerebral ischemia/reperfusion (IR) injury" OR "cerebral ischemic reperfusion (IR) injury" OR "cerebral ischemic reperfusion injury" OR "cerebral reperfusion injury" OR "reperfusion brain injury" OR "reperfusion cerebral injury" OR "reperfusion−induced brain injury" OR "reperfusion−induced cerebral injury" OR "cerebral artery occlusion" OR "brain arterial occlusion" OR "brain arteries occlusion" OR "brain artery obstruction" OR "occlusive cerebrovascular disease" OR "brain artery occlusion" OR "cerebral arterial obstruction" OR "cerebral arterial occlusion" OR "cerebral arteries occlusion" OR "cerebral artery obstruction" OR "obstruction of the cerebral arteries" OR "obstruction of the cerebral artery" OR "occlusion of the brain arteries" OR "occlusion of the cerebral arteries" OR "occlusion of the cerebral artery".

Supplement 2: Risk of bias assessment for the included studies

Item	Questions	Comments	Demyanenko 2020	Guo 2021	Demyanenko 2019	Han 2024	Wang 2016	Sheu 2021	Yang 2023
**1**	Did authors state that rodent models accounted for common risk factors?	Scored as 'yes' if rodent models had at least 1 of older age, hypertension, diabetes or comorbid atherosclerosis.	N	N	N	N	N	N	N
**2**	Did authors provide a sample size calculation to inform study design?		N	N	N	Y	N	N	N
**3**	Did authors provide a statement regarding compliance with animal welfare regulations?	Scored as 'yes' if authors stated approval by an animal ethics research committee AND detailed which code of conduct was adhered to.	Y	Y	Y	Y	Y	Y	Y
**4**	Was a statement of potential conflicts of interest provided?		Y	Y	Y	Y	Y	Y	Y
**5**	Did the study include both male and female animals?		N	N	N	N	N	Y	N
**6**	Were the STAIR guidelines cited?		N	N	N	N	N	N	N
**7**	Was the model of AIS induction described in sufficient detail in body of text to enable readers to replicate procedure?	Scored as 'yes' if all of the following details included. Anaesthesia approach, method of AIS induction (e.g. filament type/size used for MCAO) or location of thrombosis (if thrombotic model)), and duration of ischaemia (if transient).	N	Y	N	N	N	Y	Y
**8**	Did studies use monitoring tools to ensure that animals experienced significant reductions in cerebral blood flow upon AIS induction, OR specify that animals must exhibit neurological deficits for inclusion? And did the study use monitoring of temperature to ensure temperature was maintained during AIS induction?	E.g. use of transcranial laser dopplers, neurological functional tests with a defined threshold that must be met, etc.	N	N	N	Y*	N	N	Y
**9**	Did the authors state that animals were randomised to treatment groups?		N	N	N	Y	Y	N	Y
**10**	Was the outcome assessor blinded to group allocations.		N	Y	N	N	N	N	Y
**11**	Did the authors report the inter/intra-observer reproducibility of key outcome measures?		N	N	N	N	N	N	N
**12**	Was the HDAC inhibitor dose and method of administration described?	Scored as 'yes' if all of the following are provided: An indication of dose amount, route and frequency of administration and time of administration relative to AIS induction.	Y	N	Y	N	Y	Y	N
**13**	Did the authors demonstrate that the agent to inhibited HDAC6 activity in vivo	e.g Histone acetylation assessment, HDAC protein expression, HDAC activity assay	Y	N	Y	N	Y	Y	Y
**Total Score out of 13**			3 (23.1%)	4 (30.8%)	4 (30.8%)	5 (38.5%)	5 (38.5%)	6 (46.2%)	7 (53.8%)
**Overall risk of bias**			High	High	High	Medium	Medium	Medium	Low

* Han *et al.* (2024) made reference to contralateral lower limb paralysis indicating successful AIS induction although the relevance of this to the conducted experiments was not enunciated. For the purposes of the current review, this statement has been interpreted to mean that this was used as a quality control indicator of AIS induction.

Supplement 3A: Cerebral infarction data used for meta-analysis using data from all studies

Study	Days post-AIS	No. doses	HDAC6i timing	HDAC6 inhibitor	Species	Model	Units of measurement	Intervention group				Control group			
								Mean	SEM	N	SD	Mean	SEM	N	SD
Demyanenko (2019)	4	2	Delayed	TubA 25mg/kg	Mouse	PTI	mm^3	13.4	3.1	8	8.8	22.5	3.4	8	9.6
Demyanenko (2019)	7	2	Delayed	TubA 25mg/kg	Mouse	PTI	mm^3	7.3	1.6	8	4.5	13.9	2.8	8	7.9
Demyanenko (2020)	7	?	Rapid	HPOB 10mg/kg	Mouse	PTI	mm^3	10.8	2.2	7	5.8	26.6	3.2	7	8.5
Wang (2016) expt 1	3	4	Rapid	TubA 25mg/kg	Rat	MCAO	mm^3	96	20	8	56.6	246	40	4	113.1 [↑]
Wang (2016) expt 1	3	4	Rapid	TubA 40mg/kg	Rat	MCAO	mm^3	90	30	8	84.9	246	40	4	113.1 [↑]
Wang (2016) expt 2	3	3	Delayed	TubA 25mg/kg	Rat	MCAO	mm^3	145.9	24.3	8	68.7	251.5	30.4	8	86.0
Guo (2021)	1	1	Rapid	TubA 25mg/kg	Rat	MCAO	% brain area	13	3	8 [α]	8.5	22.1	2.1	3	5.9 [α↑]
Guo (2021)	1	1	Rapid	Compound 5	Rat	MCAO	% brain area	10.2	2.3	8 [α]	6.5	22.1	2.1	3	5.9 [α↑]
Guo (2021)	1	1	Rapid	Compound 18	Rat	MCAO	% brain area	11.8	3	8 [α]	8.5	22.1	2.1	2	5.9 [α↑]
Han (2024)	1	1	Rapid	Compound 3	Rat	MCAO	% brain area	32.3	13.9	5	31.1	49.9	8.1	5	18.1
									**Total:**	**76**			**Total:**	**52**	

Days post-AIS refers to the timing of that brain infarction volume assessment relative to AIS induction. No. doses refers to the number of times that HDAC6 inhibitor or control was administered during the experiment. HDAC6i timing refers to whether the animals received intervention rapidly (within 1 hour of PTI or upon reperfusion for MCAO models), or after a delay (≥24 hours) following AIS onset. SEM: standard error of mean. SD: Standard deviation (calculated as SEM x √total group size) TubA: Tubastatin A. HPOB: 4-[(Hydroxyamino)carbonyl]-N-(2-hydroxyethyl)-N-phenyl-benzeneacetamide. PTI: Photothrombosis. MCAO: Middle cerebral artery occlusion.

α Data presented in the paper by Guo *et al.* indicate that groups contained seven or eight animals although exact numbers were not specified. For the purposes of this analysis, group sizes are standardised to include 8 animals.

↑ These studies compared multiple experimental groups to a common control group. To avoid over-estimating the assessed population analysed, the number of animals in the original control groups were split into equal groups with the same mean and standard error. The total size of the control group for each study (used to calculate standard deviation) is the sum of the constituent subgroups.

Supplement 3B: Cerebral infarction data used for meta-analysis using data from studies using commercially available HDAC6 inhibitors

Study	Days post-AIS	No. doses	HDAC6i timing	HDAC6 inhibitor	Species	Model	Units of measurement	Intervention group				Control group			
								Mean	SEM	N	SD	Mean	SEM	N	SD
Demyanenko (2019)	4	2	Delayed	TubA 25mg/kg	Mouse	PTI	mm^3	13.4	3.1	8	8.8	22.5	3.4	8	9.6
Demyanenko (2019)	7	2	Delayed	TubA 25mg/kg	Mouse	PTI	mm^3	7.3	1.6	8	4.5	13.9	2.8	8	7.9
Demyanenko (2020)	7	?	Rapid	HPOB 10mg/kg	Mouse	PTI	mm^3	10.8	2.2	7	5.8	26.6	3.2	7	8.5
Wang (2016) expt 1	3	4	Rapid	TubA 25mg/kg	Rat	MCAO	mm^3	96	20	8	56.6	246	40	4	113.1 ↑
Wang (2016) expt 1	3	4	Rapid	TubA 40mg/kg	Rat	MCAO	mm^3	90	30	8	84.9	246	40	4	113.1 ↑
Wang (2016) expt 2	3	3	Delayed	TubA 25mg/kg	Rat	MCAO	mm^3	145.9	24.3	8	68.7	251.5	30.4	8	86.0
Guo (2021)	1	1	Rapid	TubA 25mg/kg	Rat	MCAO	% brain area	13	3	8 α	8.5	22.1	2.1	8	5.9 α ↑
								**Total:**		**55**		**Total:**		**47**	

Days post-AIS refers to the timing of that brain infarction volume assessment relative to AIS induction. No. doses refers to the number of times that HDAC6 inhibitor or control was administered during the experiment. HDAC6i timing refers to whether the animals received intervention rapidly (within 1 hour of PTI or upon reperfusion for MCAO models), or after a delay (≥24 hours) following AIS onset.SEM: standard error of mean. SD: Standard deviation (calculated as SEM x √total group size) TubA: Tubastatin A. HPOB: 4-[(Hydroxyamino)carbonyl]-N-(2-hydroxyethyl)-N-phenyl-benzeneacetamide. PTI: Photothrombosis. MCAO: Middle cerebral artery occlusion. α Data presented in the paper by Guo *et al.* indicate that groups contained seven or eight animals although exact numbers were not specified. For the purposes of this analysis, group sizes are standardised to include 8 animals.

↑ These studies compared multiple experimental groups to a common control group. To avoid over-estimating the assessed population analysed, the number of animals in the original control groups were split into equal groups with the same mean and standard error. The total size of the control group for each study (used to calculate standard deviation) is the sum of the constituent subgroups.

Supplement 3C: Cerebral infarction data used for meta-analysis from studies using Tubastatin A (25 mg/kg).

Study	Days post-AIS	No. doses	HDAC6i timing	HDAC6 inhibitor	Species	Model	Units of measurement	Intervention group				Control group			
								Mean	SEM	N	SD	Mean	SEM	N	SD
Demyanenko (2019)	4	2	Delayed	TubA 25mg/kg	Mouse	PTI	mm^3	13.4	3.1	8	8.8	22.5	3.4	8	9.6
Demyanenko (2019)	7	2	Delayed	TubA 25mg/kg	Mouse	PTI	mm^3	7.3	1.6	8	4.5	13.9	2.8	8	7.9
Wang (2016) expt 1	3	4	Rapid	TubA 25mg/kg	Rat	MCAO	mm^3	96	20	8	56.6	246	40	8	113.1
Wang (2016) expt 2	3	3	Delayed	TubA 25mg/kg	Rat	MCAO	mm^3	145.9	24.3	8	68.7	251.5	30.4	8	86.0
Guo (2021)	1	1	Rapid	TubA 25mg/kg	Rat	MCAO	% brain area	13	3	8 α	8.5	22.1	2.1	8	5.9 α
								**Total:**		**40**		**Total:**		**40**	

Days post-AIS refers to the timing of that brain infarction volume assessment relative to AIS induction. SEM: standard error of mean. SD: Standard deviation (calculated as SEM x √total group size) TubA: Tubastatin A. HPOB: 4-[(Hydroxyamino)carbonyl]-N-(2-hydroxyethyl)-N-phenyl-benzeneacetamide. PTI: Photothrombosis. MCAO: Middle cerebral artery occlusion.

α Data presented in the paper by Guo *et al.* indicate that groups contained seven or eight animals although exact numbers were not specified. For the purposes of this analysis, group sizes are standardised to include 8 animals.

↑ These studies compared multiple experimental groups to a common control group. To avoid over-estimating the assessed population analysed, the number of animals in the original control groups were split into equal groups with the same mean and standard error. The total size of the control group for each study (used to calculate standard deviation) is the sum of the constituent subgroups.

Supplement 4. Funnel plots, trim and fill analyses and leave one sensitivity analysis of infarct data considering data from all studies.

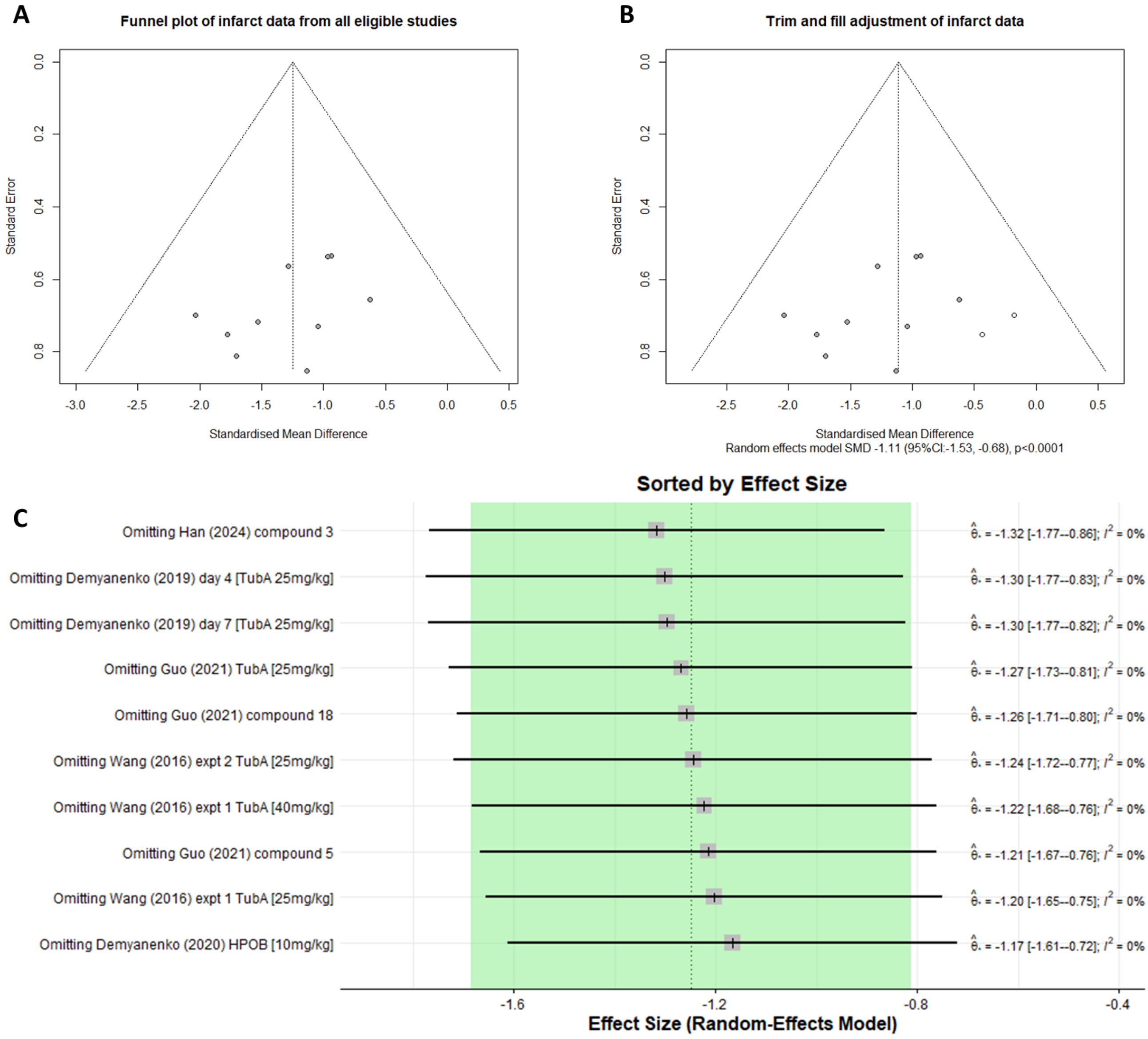

A) Funnel plot assessing publication bias following meta-analysis of data investigating the impact of HDAC6 inhibition on cerebral infarction size. Plot suggests no significant asymmetry attributable to publication bias (Egger's test – intercept: -1.87 (95% CI:-4.23, -1.55), p=0.160).

B) Outcomes of trim and fill analysis imputing 2 studies to ensure funnel plot symmetry (imputed datasets shown as unfilled bubbles). Random effects analysis incorporating imputed datasets confirms significant reduction in cerebral infarction volume in animals receiving HDAC6 inhibitors (standard mean difference: -1.11 (95% CI: -1.53, -0.68), p<0.0001).

C) Outcomes from leave-one out analyses demonstrating the effect of omitting single studies on overall effect size.

Supplement 5. Forest plots detailing impact of HDAC6 inhibition on cerebral infarction size when considering data from experiments using commercially available HDAC6 inhibitors.

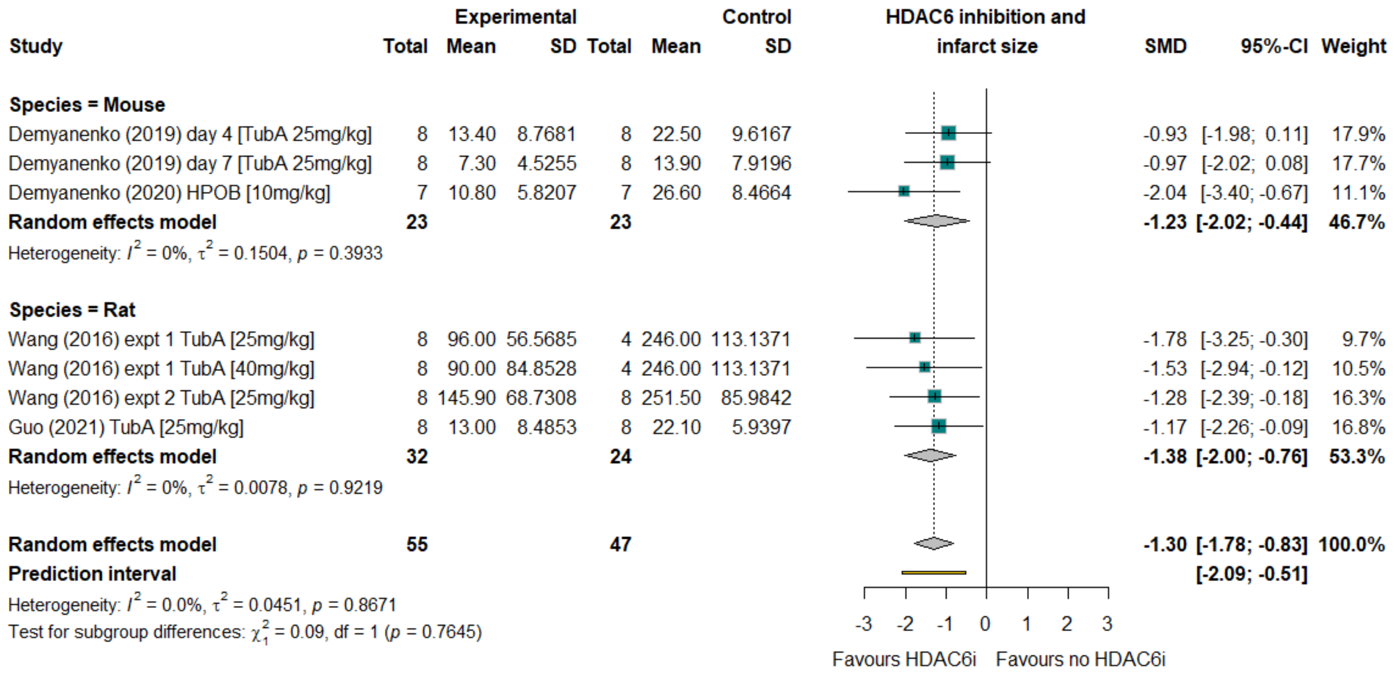

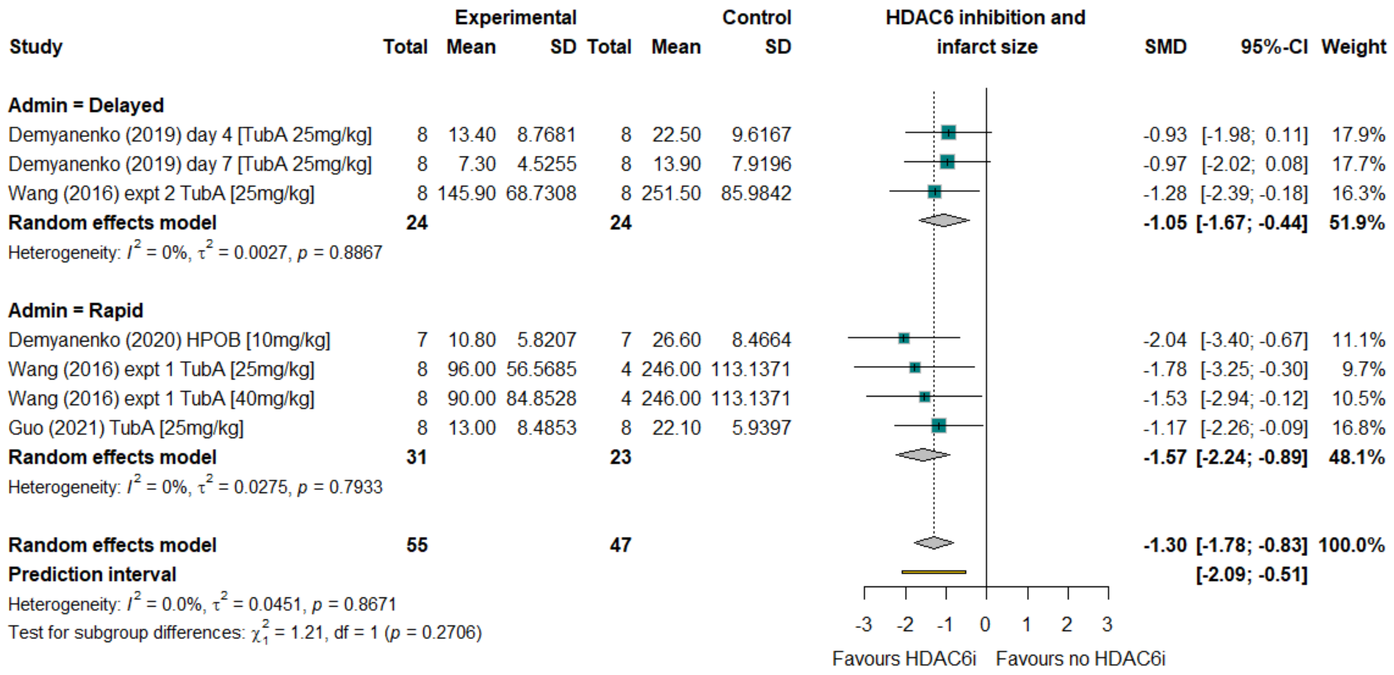

Studies are grouped to show the effect of commercial HDAC6 inhibitors on cerebral infarction size according to the model species assessed (top panel), or the time of administration relative to AIS induction (bottom panel).

Supplement 6. Forest plots detailing impact of HDAC6 inhibition on cerebral infarction size when considering data from experiments using 25 mg/kg Tubastatin A to inhibit HDAC6.

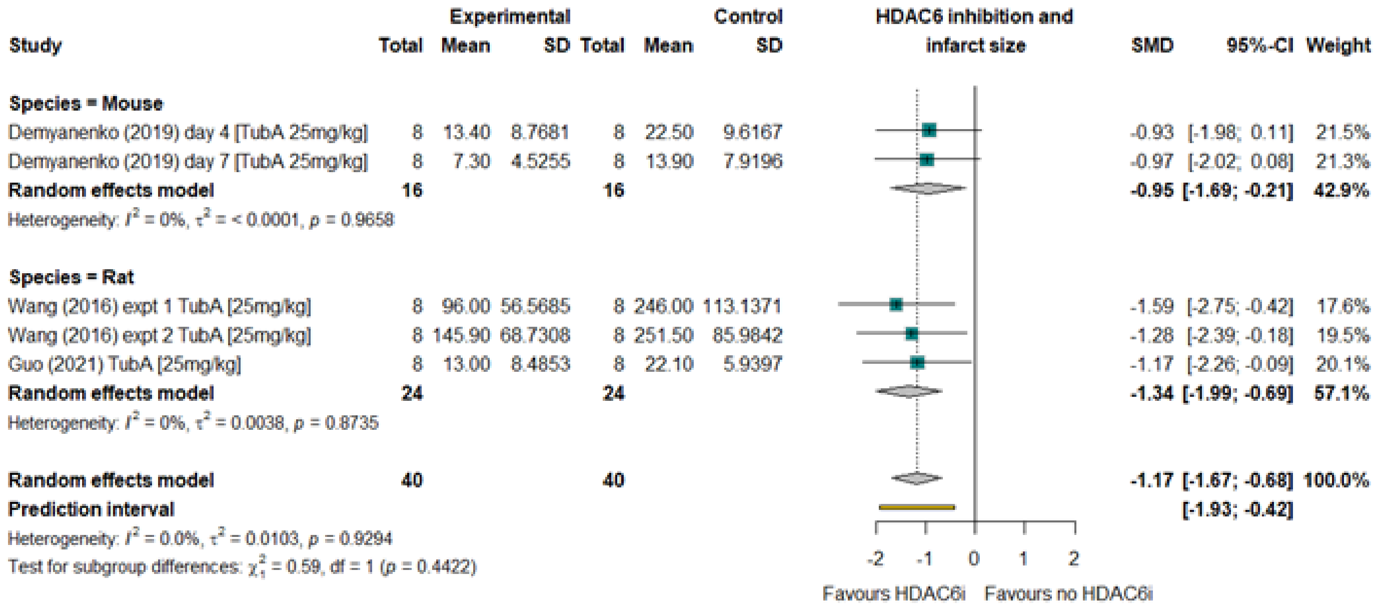

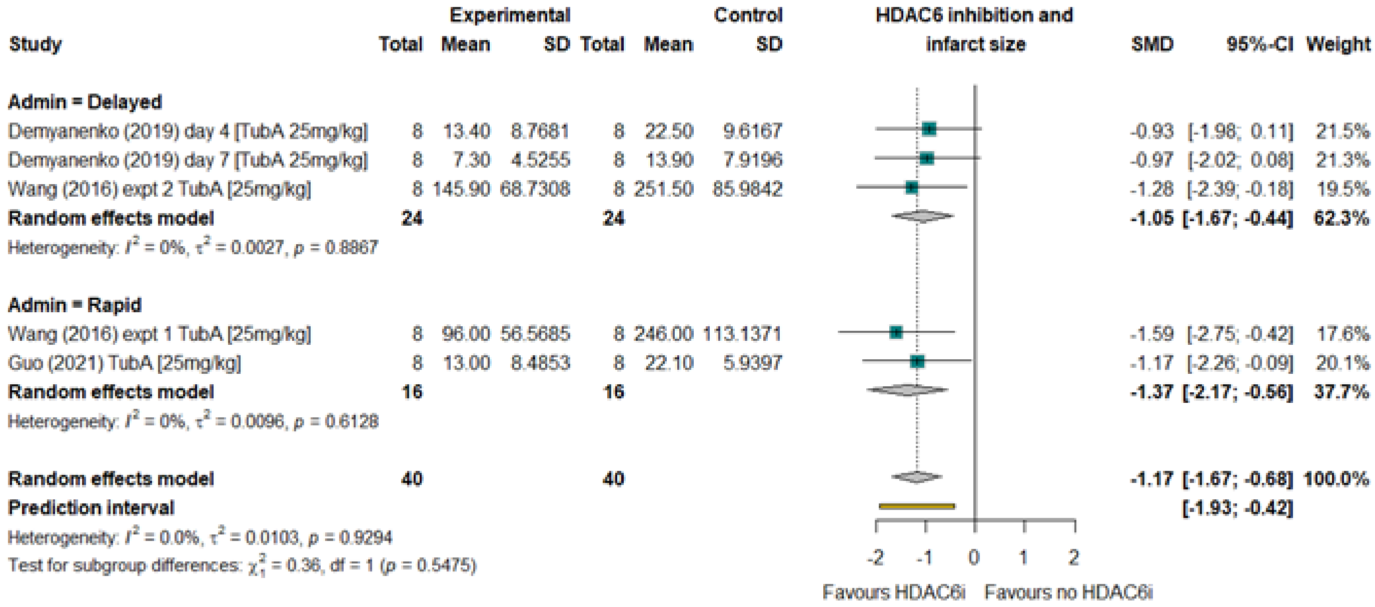

Studies are grouped to show the effect of commercial HDAC6 inhibitors on cerebral infarction size according to the model species assessed (top panel), or the time of administration relative to AIS induction (bottom panel).

Supplement 7: Outcomes of meta-regression assessing the relationship of the time of tissue harvest (A) and number of drug doses administered (B) with the difference in cerebral infarction size between animals receiving HDAC6 inhibitors or control interventions.

A

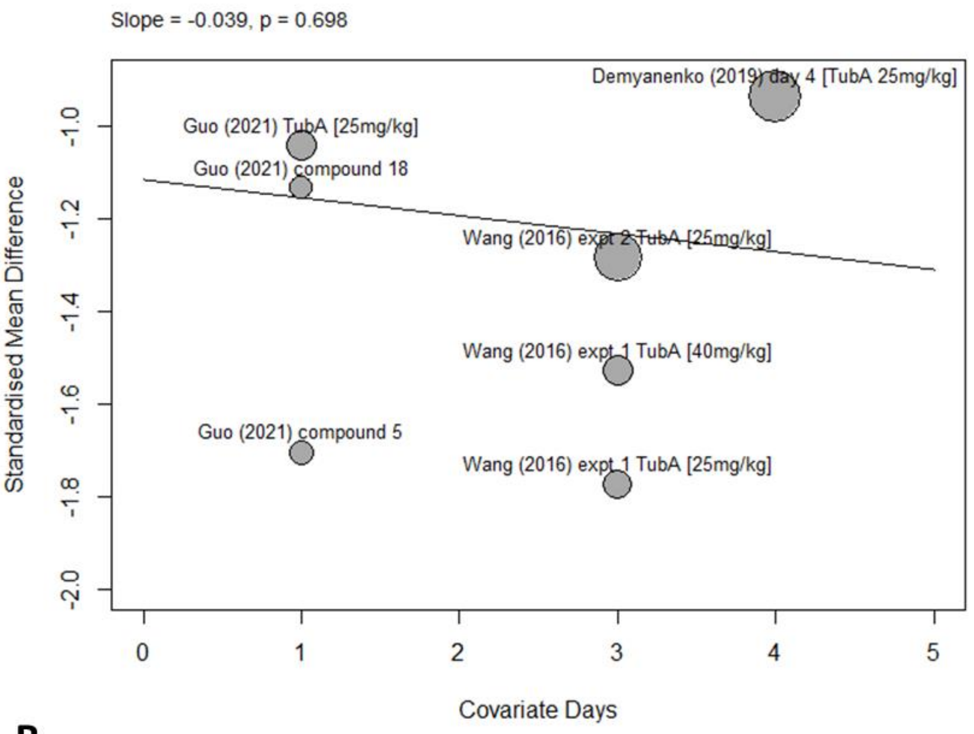

B

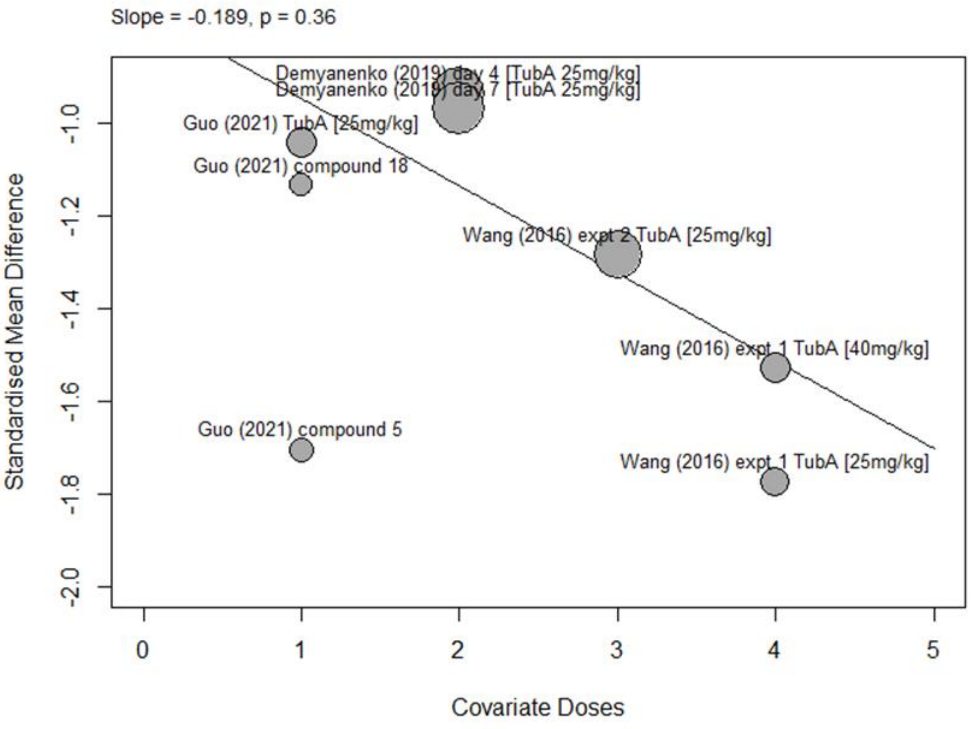

Supplement 8: Modelling the recovery of left forelimb function as reported by Demyanenko *et al.* (2019 and 2020).

Supplement 8a: Reported vs simulated data for Demyanenko cylinder test analyses

Group	Reported values		Simulated values	
	**Mean**	**SEM**	**Mean**	**SEM**
Demyanenko et al. 2019				
Control day 4	21.3	4.1	22.2	3.7
Control day 7	24.5	3.9	24.9	3.6
Control day 14	39.5	7.7	41.0	6.8
HDAC6i day 4	24.2	5.3	26.0	4.6
HDAC6i day 7	39.7	4.6	39.7	4.4
HDAC6i day 14	47.2	6.5	47.4	6.1
Demyanenko et al. 2020				
Control day 4	21.2	5.0	23.1	4.3
Control day 7	26.6	2.9	26.6	2.8
Control day 14	27.6	3.2	27.6	3.1
HDAC6i day 4	20.1	5.0	22.3	4.2
HDAC6i day 7	33.6	5.2	34.1	4.9
HDAC6i day 14	55.1	4.5	55.2	4.4

Supplement 8b: Comparing left forelimb recovery in control groups reported by Demyanenko 2019 and 2020.

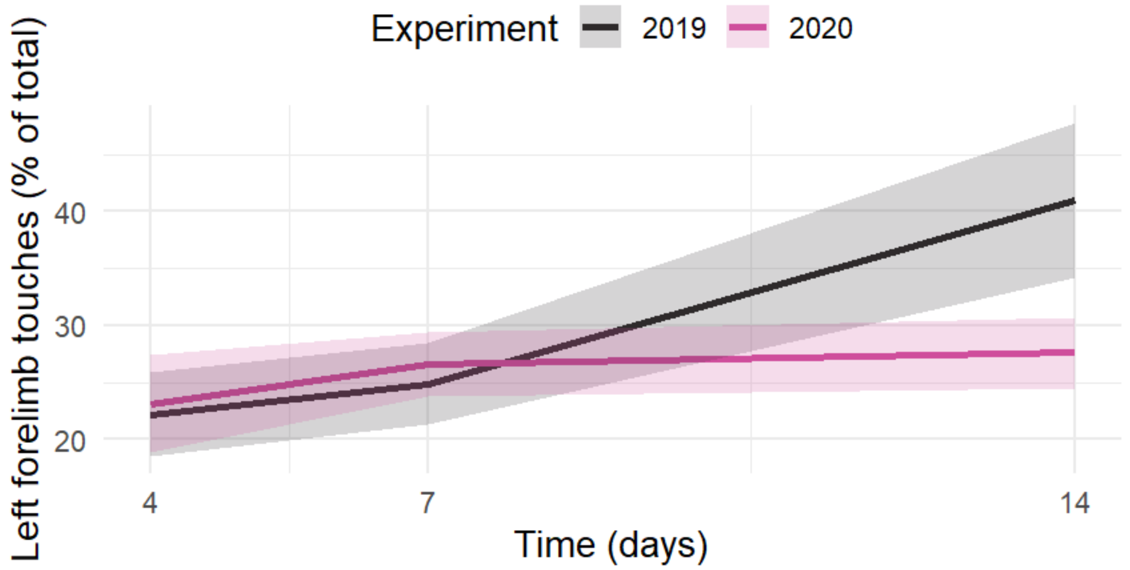

Supplement 9: Reported vs simulated data for functional outcomes reported by Wang *et al.* (2016)

Group	Rotarod				Neurological score				Tilting percentage			
	Reported values		Simulated values		Reported values		Simulated values		Reported values		Simulated values	
	Mean	SEM	Mean	SEM	Mean	SEM	Mean	SEM	Mean	SEM	Mean	SEM
Experiment 1 – HDAC6 inhibition upon reperfusion												
Control day 0	135.6	15.1	136.0	14.5	0.0	0.0	0.0*	0.0*	0.0	0.0	0.0*	0.0*
Control day 1	53.1	10.3	55.4	9.2	6.1	0.6	5.3	0.4	79.2	14.2	61.8	8.5
Control day 2	55.6	10.0	57.2	9.1	6.2	0.9	4.7	0.6	73.3	7.3	69.4	5.9
Control day 3	55.3	8.2	55.8	7.7	5.1	0.9	4.3	0.6	63.3	6.4	62.3	5.9
TubA 25 mg/kg day 0	130	8.2	130.0	8.0	0	0	0.0*	0.0*	0	0	0.0*	0.0*
TubA 25 mg/kg day 1	112.8	12.1	113.0	11.6	2.9	0.6	3.0	0.5	44.4	8.7	45.7	7.4
TubA 25 mg/kg day 2	107.8	10.6	108.0	10.3	2.3	0.4	2.4	0.4	46.1	4.8	46.1	4.6
TubA 25 mg/kg day 3	110.7	11.8	111.0	11.3	2.1	0.4	2.2	0.4	37.2	6.4	38.1	5.9
TubA 40 mg/kg day 0	125.4	8.1	125.0	7.8	0.0	0.0	0.0*	0.0*	0.0	0.0	0.0*	0.0*
TubA 40 mg/kg day 1	73.6	7.1	73.7	6.9	3.5	0.7	3.5	0.6	44.4	10.2	46.3	8.1
TubA 40 mg/kg day 2	76.7	10.1	77.0	9.6	2.9	0.6	3.0	0.5	36.3	13.0	43.5	8.6
TubA 40 mg/kg day 3	98	14.2	98.7	13.4	2.5	0.8	2.9	0.6	35.6	11.2	41.6	8.2
Experiment 2 – HDAC6 inhibition 24 hours post-reperfusion												
Control day 0	141.0	15.0	141.0	14.5	0.0	0.0	0.0*	0.0*	0.0	0.0	0.0*	0.0*
Control day 1	45.0	6.1	45.3	5.8	6.6	0.2	6.4	0.2	84.4	10.9	69.1	7.1
Control day 2	54.0	6.0	54.0	5.8	6.7	0.6	5.6	0.4	72.5	5.7	70.9	5.0
Control day 3	58.5	9.0	59.3	8.4	5.7	0.6	5.1	0.4	61.3	5.2	61.1	5.0
TubA 25 mg/kg day 0	136.6	10.7	137.0	10.4	0.0	0.0	0.0*	0.0*	0.0	0.0	0.0*	0.0*
TubA 25 mg/kg day 1	51.1	3.4	51.1	3.3	6.3	0.3	6.0	0.3	81.3	4.3	79.7	3.7
TubA 25 mg/kg day 2	95.9	11.1	95.9	10.7	4.6	0.4	4.6	0.4	53.1	8.9	52.4	7.6
TubA 25 mg/kg day 3	102.4	14.1	103.0	13.3	4.1	0.5	4.1	0.5	43.1	7.4	43.9	6.7
Merged controls												
Merged controls day 0	---	---	138.0	10.4	---	---	---	---	---	---	---	---
Merged controls day 1	---	---	50.3	5.7	---	---	---	---	---	---	65.5	5.7
Merged controls day 2	---	---	55.6	5.5	---	---	---	---	---	---	70.2	4.0
Merged controls day 3	---	---	57.5	5.8	---	---	---	---	---	---	61.7	3.90

* Simulated baseline data were not included in the modelling analyses as these denote performance in otherwise healthy animals. Models included data from days 1-3 in order to assess differences in the rates of recovery between experimental groups.

Supplement 9A: Comparing longitudinal outcomes between control groups in separate experiments reported by Wang *et al.* (2016).

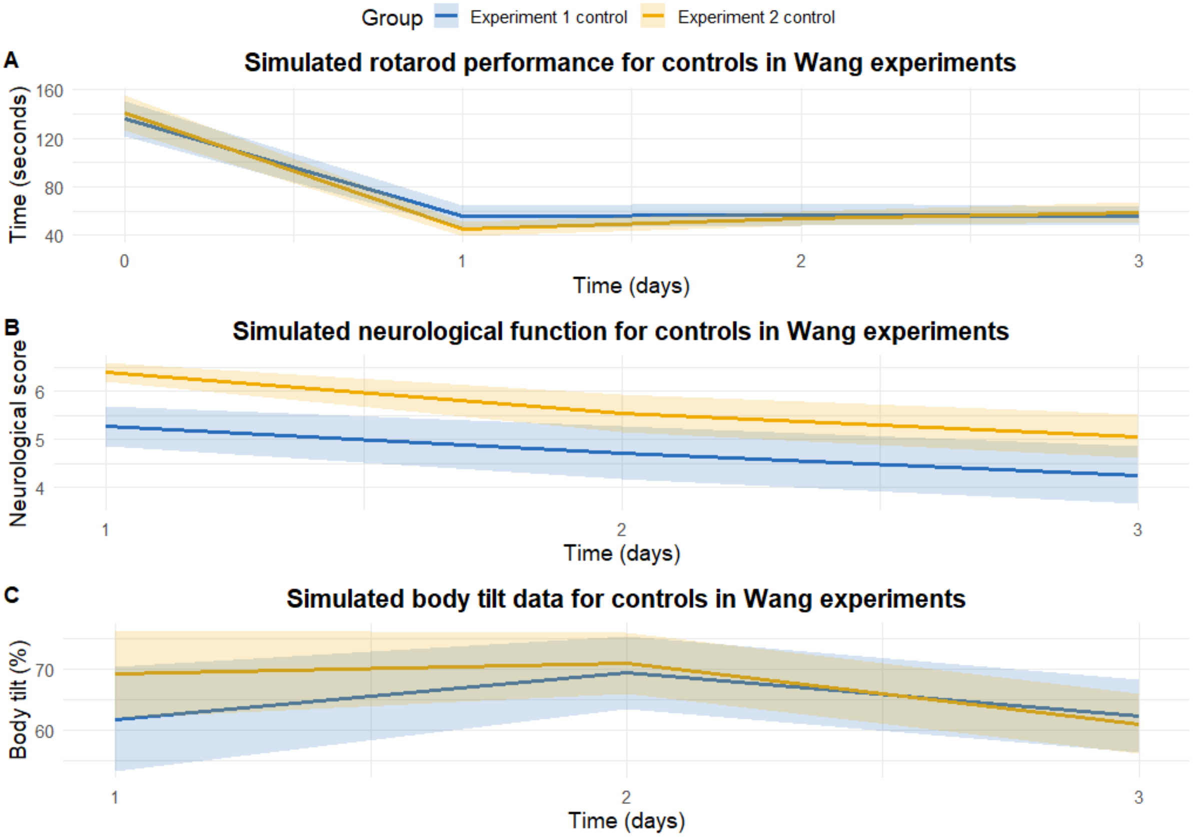

Shown are graphs detailing the simulated data for control groups from independent experiments reported by Wang *et al.* (2016). Data summarise means (solid lines) and standard errors (halos) from 20,000 simulated datasets. Data from Rotarod (A) and body tilt (C) experiments were considered compatible for meta-analysis evidenced by clear longitudinal overlap in the performance of the groups. Neuroscore outcomes (B) showed minimal longitudinal overlap indicating that the data were not suitable for meta-analysis. Note, baseline data for the Neuroscore and body tilt outcomes were not included in the modelling analyses and are therefore not shown here.
